# The Theta Paradox: 4-8 Hz EEG Oscillations Reflect Both Sleep Pressure and Cognitive Control

**DOI:** 10.1523/JNEUROSCI.1063-22.2022

**Published:** 2022-11-09

**Authors:** Sophia Snipes, Elena Krugliakova, Elias Meier, Reto Huber

**Affiliations:** ^1^Child Development Center, University Children's Hospital Zürich, University of Zürich, 8032 Zürich, Switzerland; ^2^Neural Control of Movement Lab, Department of Health Sciences and Technology, ETH Zürich, 8092 Zürich, Switzerland; ^3^Department of Child and Adolescent Psychiatry and Psychotherapy, Psychiatric Hospital, University of Zürich, 8008 Zürich, Switzerland

**Keywords:** EEG, frontal midline, local sleep, short term memory, sleep deprivation, theta

## Abstract

Human electroencephalographic (EEG) oscillations characterize specific behavioral and vigilance states. The frequency of these oscillations is typically sufficient to distinguish a given state; however, theta oscillations (4–8 Hz) have instead been found in near-opposite conditions of drowsiness during sleep deprivation and alert cognitive control. While the latter has been extensively studied and is often referred to as “frontal midline theta,” (fmTheta) the former has been investigated far less but is considered a marker for sleep pressure during wake. In this study we investigated to what extent theta oscillations differed during cognitive tasks and sleep deprivation. We measured high-density EEG in 18 young healthy adults (nine female) performing six tasks under three levels of sleep deprivation. We found both cognitive load and sleep deprivation increased theta power in medial prefrontal cortical areas; however, sleep deprivation caused additional increases in theta in many other, predominantly frontal, areas. The sources of sleep deprivation theta (sdTheta) were task dependent, with a visual-spatial task and short-term memory (STM) task showing the most widespread effects. Notably, theta was highest in supplementary motor areas during passive music listening, and highest in the inferior temporal cortex (responsible for object recognition) during a spatial game. Furthermore, while changes in task performance were correlated with increases in theta during sleep deprivation, this relationship was not specific to the EEG of the same task and did not survive correction for multiple comparisons. Altogether, these results suggest that both during sleep deprivation and cognition theta oscillations may preferentially occur in cortical areas not involved in ongoing behavior.

**SIGNIFICANCE STATEMENT** Electroencephalographic (EEG) research in sleep has often remained separate from research in cognition. This has led to two incompatible interpretations of the function of theta brain oscillations (4–8 Hz): that they reflect local sleep events during sleep deprivation, or that they reflect cognitive processing during tasks. With this study, we found no fundamental differences between theta oscillations during cognition and theta during sleep deprivation that would suggest different functions. Instead, our results indicate that in both cases, theta oscillations are generated by cortical areas not required for ongoing behavior. Therefore, at least in humans, theta may reflect either cortical disengagement or inhibition.

## Introduction

Oscillations in the electroencephalography (EEG) have been associated with behavioral states such as alertness, drowsiness, and sleep. This typically allows oscillations to be used as objective markers for vigilance. The exception are theta oscillations (4–8 Hz), which have been separately identified as indicators of drowsiness and intense cognition.

Theta oscillations increase during sleep deprivation in animals (Vyazovskiy and Tobler, 2005) and humans (Aeschbach et al., 1997). Theta is considered to reflect sleep pressure, i.e., the interaction between circadian rhythm and time spent awake determining when an individual feels the need to sleep (Borbely, 1982; Cajochen et al., 2001). Given the presence of theta oscillations when and where sleep pressure is highest (Finelli et al., 2000), they have been hypothesized to be a form of local sleep during wake (Vyazovskiy et al., 2011; Siclari and Tononi, 2017). During sleep, slow waves (0.5–4 Hz) in the surface EEG correspond to synchronized silencing of neuronal spiking, known as “off periods” (Steriade et al., 2001). Vyazovskiy et al. (2011) found these off periods to also occur during sleep deprived awake rats, corresponding to theta oscillations in local field potentials.

Equally robust research has separately linked theta activity to cognition. Theta has been associated with a variety of functions (Buzsáki, 2005), most notably hippocampal theta during spatial navigation in rats (O'Keefe and Recce, 1993; Buzsáki, 1996) and frontal-midline theta (fmTheta) during cognitive tasks in humans. fmTheta has been associated with arithmetic (Ishihara and Yoshii, 1967; Ishii et al., 2014), working memory (Gevins et al., 1998; Jensen and Tesche, 2002), and even meditation (Banquet, 1973; D.J. Lee et al., 2018). fmTheta has been source-localized to the anterior cingulate cortex and medial prefrontal cortex (Onton et al., 2005; Michels et al., 2010; Ishii et al., 2014), where it has been anti-correlated to functional magnetic resonance imaging, blood-oxygen level-dependent (fMRI BOLD) activity in these areas (Scheeringa et al., 2008, 2009). The exact function of fmTheta oscillations in cognition is still unresolved although various explanations have been proposed (Klimesch et al., 2005; Sauseng et al., 2010; Hsieh and Ranganath, 2014). One of the most well-elaborated hypotheses is that theta is responsible for synchronizing neuronal firing across cortical regions (Lisman and Jensen, 2013). This has been supported by intracortical recordings in macaques for short-term memory tasks (H. Lee et al., 2005; Liebe et al., 2012). Evidence in humans has been mixed (Brzezicka et al., 2019); however, given the strong association with tasks, theta is generally hypothesized to be functionally relevant for cognitive processing.

Currently, research in theta oscillations increasing with sleep deprivation (sdTheta; Finelli et al., 2000; Vyazovskiy et al., 2011) has remained largely independent from research in cognition and fmTheta (Jensen and Tesche, 2002; Ishii et al., 2014; Maurer et al., 2015). It is therefore unknown whether these represent either two distinct oscillations in the theta range or the same, as has been suggested by Takahashi et al. (1997) and Mitchell et al. (2008). If sdTheta and fmTheta are distinct, this would resolve the apparent paradox of an oscillation reflecting both drowsiness and cognition. If sdTheta is instead a manifestation of fmTheta, then its interpretation as local sleep should be reconsidered.

We conducted this exploratory sleep deprivation study in young healthy adults to disentangle the changes in theta related to both drowsiness and cognition using high-density EEG. Six tasks were performed under three levels of sleep pressure. To determine whether sdTheta and fmTheta could be considered the same oscillation, we first looked at their topography within a short-term memory task and source-localized their neural substrates. We also inspected their spectrograms to determine whether they could be differentiated by peak frequency. To explore more generally whether sdTheta is affected by behavioral state, we compared its topography and source localization in all six tasks. Lastly, to determine what impact sdTheta and fmTheta might have, we correlated changes in theta with changes in behavioral performance.

## Materials and Methods

### Participants

Participants were recruited from Swiss universities through online advertisements and word of mouth and screened for eligibility with an online questionnaire. Out of 75 applicants, one was recruited for technical pilots (data not included), 31 passed but did not initiate contact or were unable to meet the scheduling requirements, 19 participants were recruited, and one dropped out midway and so was not included in further analyses. Of the 18 participants who completed the experiment, nine were female and three were left-handed. Mean age (±standard deviation) was 23 ± 1 years old. All participants self-reported above-average English fluency (68 ± 13% on a scale from “terrible” to “native speaker”), with one participant a native English speaker. All had corrected-to-normal vision and self-reported no hearing impairments.

Applicants were screened before participating to: (A) have a uniform, neurotypical population; (B) avoid potential drop-outs because of adverse reactions to the experimental conditions; (C) ensure participants' lifestyles were similar enough to the requirements of the control week (the week before each recording session) so as not to cause major disruptions; (D) avoid any health or life conditions that could interact negatively with sleep deprivation or other experimental conditions; (E) ensure participants were at least somewhat vulnerable to sleep deprivation to avoid floor effects.

Inclusion criteria were:

- Age between 18 and 25 (A)
- Good sleepers, with a Pittsburgh Sleep Quality Index (PSQI) ≤ 5 (Buysse et al., 1989), few night-time awakenings, and resistance to adverse environmental conditions such as background noise or dim lights (B)
- A regular sleep-wake rhythm, with a Munich Chronotype Questionnaire (MCTQ) score between 2 and 6.5 (Roenneberg et al., 2015), sleep duration between 6 and 11 h, a preferred bedtime between 21:00-01:00 and wakeup time between 06:00-11:00 (A, C)
- A body mass index (BMI) between 18 and 30 (A, D)

Exclusion criteria were:

- Habitual napping (C)
- Sleep-related disturbances or disorders such as insomnia or daytime sleepiness (D)
- Pregnancy or currently experiencing a difficult period in life (e.g., stress, loss, etc.; D)
- Any medical, psychological, or psychiatric conditions (B, D)
- Any physical impairment at the time of recording or recent use of a long-term cast/bandage (D)
- Sensitive skin (B)
- Currently or recently taking prescription medication, excluding contraceptives (A, D)
- Regular recreational drug consumption, use of prescription stimulants, heavy consumers of alcohol (either daily consumption or occasional binge drinking), or smokers (A, C)
- Habitual consumption of more than three cups of coffee per day (C)
- Prior experience with shift work, regular experience with changing time zones, or spending > 20 h awake (E)
- Resilience to sleep deprivation (E)

Data collection and interaction with participants were conducted according to Swiss law (Ordinance on Human Research with the Exception of Clinical Trials) and the principles of the Declaration of Helsinki, with Zurich cantonal ethics approval BASEC-Nr. 2019-01193. All participants signed informed consent before participation and were made aware that they could terminate the experiment at any time. Because of scheduling restraints caused by the COVID-19 pandemic, some leniency was allowed for edge cases of the screening criteria (e.g., one participant was 26 at the time of recording, another had early morning work experience as a baker).

### Experiment design

Participants came to the laboratory twice, first for the baseline then the sleep deprivation bout, separated by at least 4 d. Experiments were typically conducted on weekends. The baseline was scheduled first to determine whether participants could in fact sleep in the laboratory and tolerate the EEG net before attempting the substantially longer sleep deprivation protocol. Data were collected between February and December 2020, overlapping with the COVID-19 pandemic and consequent lockdowns. Because of scheduling restraints, four participants conducted the baseline after the sleep deprivation recording, so the experimental session orders were not balanced, nor uniform.

During the week before each session, participants were asked to maintain a regular sleep wake cycle, going to bed and waking up within 1 h of a predetermined sleep and wakeup time based on their personal preference. These individualized sleep and wake times were then used during the experiment. During the control week, participants wore a wrist accelerometer (GENEActiv, Activinsights Ltd.) and filled out regular sleep reports to ensure compliance. Participants were further asked to abstain from alcohol in the 3 d before the measurement, and limit caffeine consumption to no more than the equivalent of two cups of coffee, and never after 16:00. They were asked to avoid time-zone travel and any activities they knew could affect their sleep (e.g., parties, skiing, sauna).

#### Baseline

**Figure 1.**
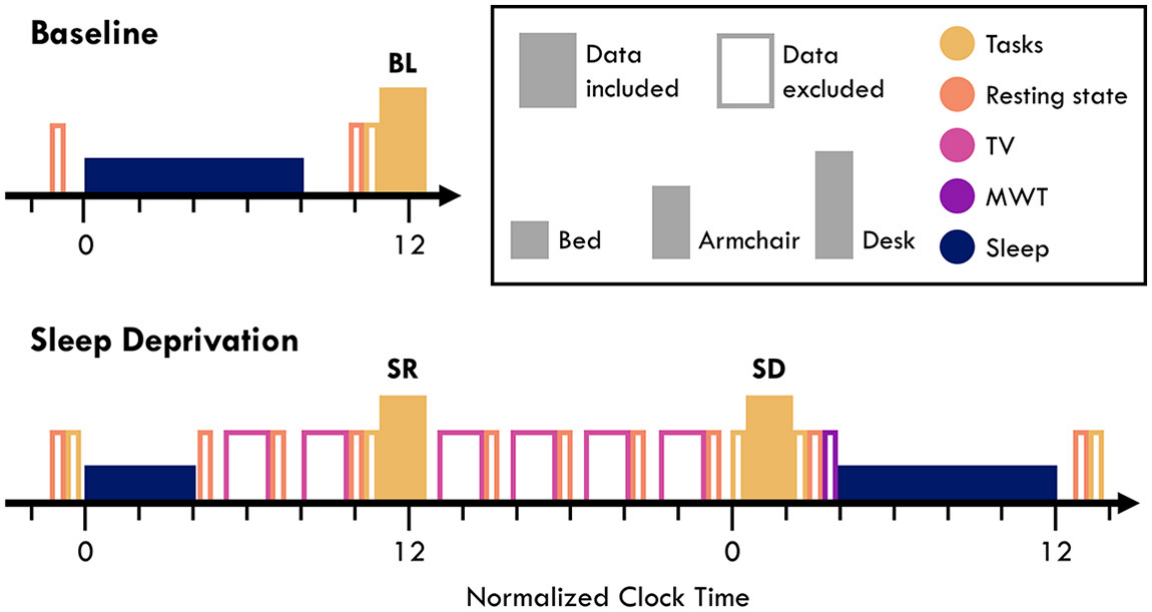
Experiment timeline. Each block indicates an EEG recording session. Filled blocks indicate data analyzed in this paper. Color indicates the activity participants engaged in: sleep (dark blue), the maintenance of wakefulness test (MWT; purple), TV watching (pink), resting state recordings (orange), and tasks (yellow). The height of each block indicates the condition in which data were collected: lying in bed (short), seated in a comfortable armchair with foot and backrest (medium), and seated at a desk (tall). The desk task block included the six tasks of this paper (STM, LAT, PVT, Speech, Game, Music) in randomized counterbalanced order, repeated three times during baseline (BL), sleep restriction (SR), and sleep deprivation (SD). The armchair task blocks included the PVT and LAT, in the same order for each participant as in the desk task block. These were counterbalanced to either come before or after the desk task block. Two additional armchair LAT recordings were performed after the SD session. Brief empty spaces indicate transition periods allowing for delays. Six longer breaks were included before each TV block in which participants were provided with meals. The exact timing was adjusted to individual habitual bedtimes, with the above diagram depicting the schedule for a bedtime of 00:00. Participants were free to wake up when they wished at baseline and during the recovery night and were woken up after 4 h during the first sleep deprivation night.

Participants first prepared for bed, then the EEG net was set up. After impedances were checked, participants were given careful instructions on how to perform the different tasks (with brief practice demonstrations), and to avoid touching the net or other movements during recordings. Afterwards, participants went to bed at the agreed-on time (21:55 to 00:47) and were left to sleep for as long as they wished (6.2–10.3 h). In the morning, participants first filled out a sleep quality questionnaire (data not included). Then, participants were provided breakfast and given time to wake up. Finally, participants performed the baseline (BL) task block (8:10–11:17), 1.8 ± 0.6 h from wake onset. Additionally, a brief resting wake recording was conducted in the evening and in the morning; however, the data were not included in this manuscript. The complete schedule is depicted in Figure 1.

#### Sleep deprivation

Participants went to bed at the same time as the baseline. They were woken up 4 h later. Throughout the day, participants repeated six cycles, each consisting of a break, two TV episodes from a series of their choice, and a brief rest recording. During the breaks, participants were provided a small home-cooked meal (selecting items from a menu beforehand), thus eating the same plate during every break. They repeated two of these cycles in the early morning, then conducted the morning sleep restriction (SR) task block after 6.4 ± 0.2 h from wake onset (within 7.7 ± 39.5 min of the BL block). The SR block was included to identify the effects of time spent awake and asleep while controlling for circadian clock time. Participants went through four more cycles before conducting the sleep deprivation (SD) task block, after 20.0 ± 0.1 h from wake onset and within 2.6 ± 10.5 min of the prior night's bedtime. Following the tasks, participants preformed a final rest test, then a maintenance of wakefulness test (MWT) in which they had to try and stay awake in a dark room for as long as possible (data not included). After 23.6 ± 0.5 h of wake, participants went to bed and slept for as long as they wished. As with the baseline bout, additional rest recordings were conducted before and after each night (data not included).

During wake recordings, participants were monitored by an experimenter to ensure they did not fall asleep. From the evening before the first night to the day after the recovery night, participants remained in the sleep laboratory and did not have access to clocks or external time cues. Two participants reported nausea with increasing sleep deprivation and were therefore provided a break outside just before the SD block (in complete nocturnal darkness).

### Tasks

Each task block lasted ∼2 h. The six tasks were performed seated upright at a desk in a well-lit room (∼100 lux at eye level), on a laptop. The order of tasks was randomized and counterbalanced across participants. For each participant, tasks were conducted in the same order for all three blocks. In addition to the main task block, two tasks [lateralized attention task (LAT), psychomotor vigilance task (PVT); see below] were performed under soporific conditions (comfortable armchair, 10-lux lighting), counterbalanced either before or after the main desk task block, as well as after the first evening and last morning rest recordings of the sleep deprivation bout (see Fig. 1). This condition is not included in this manuscript. Each task began and ended with a 1-min rest period allowing participants to adjust and get comfortable. After each task, participants answered a task battery questionnaire asking how they experienced the task.

#### Short-term memory (STM) task

**Figure 2.**
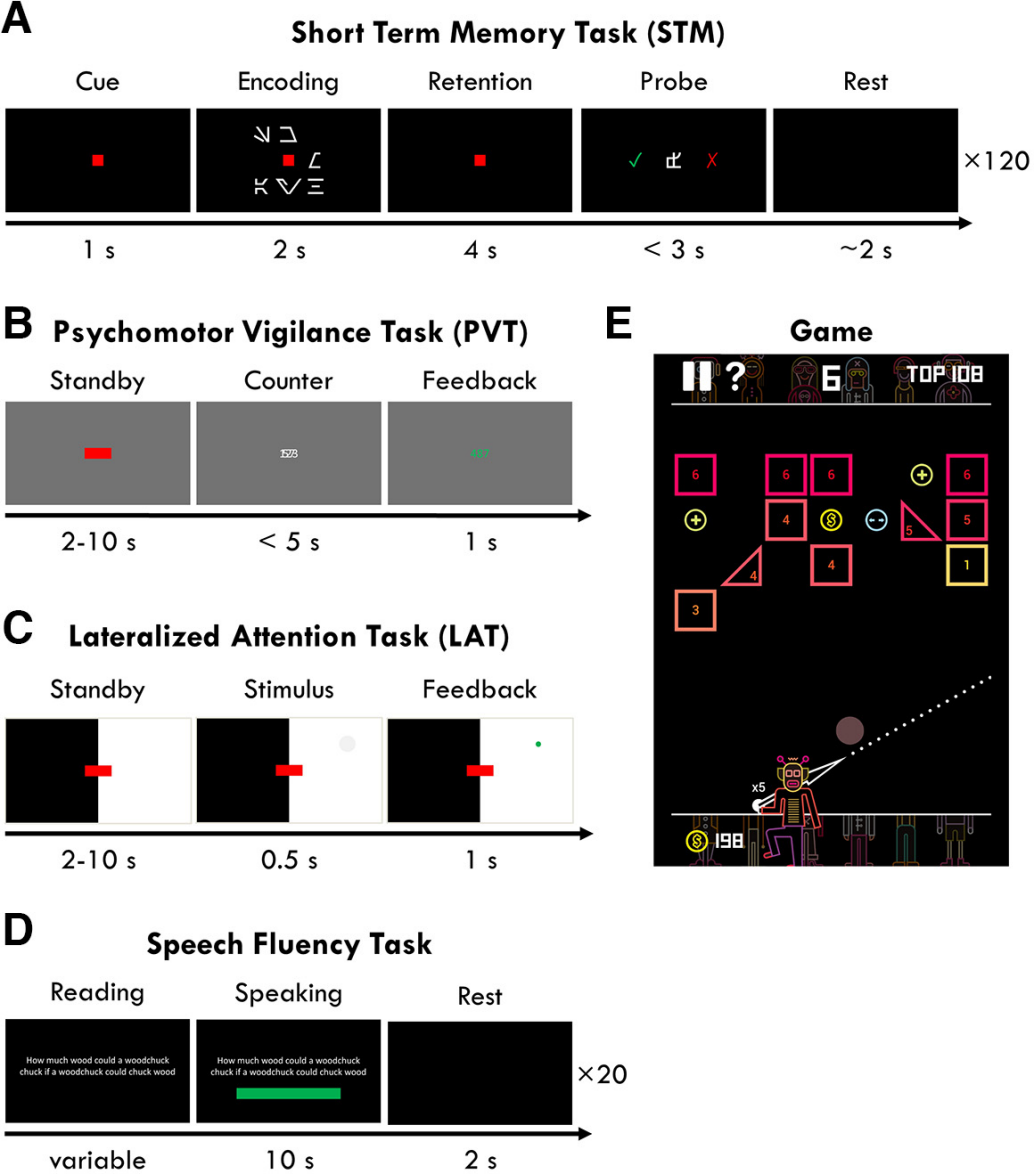
Task stimuli. ***A–D***, Tasks were performed on a Lenovo ThinkPad P53 laptop (15.6-inch FHD, Intel Core i7-9750H) with Windows 10. The computer was kept at 50% volume and 100% brightness for all tasks. The tasks were programmed in Python v3.6.5 using the PsychoPy v3.2.4 toolbox. Digital triggers were sent from the task computer to the EEG recording system via USB. Responses for the PVT and LAT were recorded with the USB-connected MilliKey button box. ***E***, The Game was played on a 10.1-inch Huawei MediaPad T5, running Android Oreo.

Participants performed a ∼25-min delayed match-to-sample/short-term memory task, adapted from Habeck et al. (2004) and Maurer et al. (2015). The task consisted of 120 trials divided in four blocks, with three memory load levels randomized across trials for a total of 40 trials per level. Stimuli are depicted in Figure 2*A*. Each trial was separated by a 1- to 2-s pause with a black screen. The encoding window began when a red fixation square appeared in the center of the screen for 1 s. Then one, three, or six symbols (selected from a pool of 30 “letters” of the Aurebesh fictional alphabet) were displayed around the fixation point in 8 possible locations for 2 s. Participants were instructed to maintain fixation on the red square while memorizing these symbols. This was followed by a 4-s retention window in which only the fixation point was displayed, and participants had to hold in memory the symbols. The trial ended with the probe window, in which a probe symbol replaced the central fixation point and participants had to indicate with left or right arrow keys whether the probe symbol was contained in the encoding set or not, within 3 s. The probe was from the encoding set in 50% of trials. No feedback on performance was provided. Accuracy was the primary outcome measure of the STM task, calculated as the percentage of correct rejections + correct acceptances to the probe relative to the total number of trials.

#### Psychomotor vigilance task (PVT)

This is a standard reaction-time task used in sleep deprivation paradigms, based on Basner and Dinges (2011). The total task duration was 10 min. Participants were presented with a red fixation rectangle on a gray background (Fig. 2*B*). Every 2–10 s, the rectangle was replaced with a millisecond countdown and participants had to press a button as fast as possible to stop it. The response time would then freeze for 1 s and be colored in yellow if less than 0.1 s (false alarm), green if between 0.1 and 0.5 s (correct response), and red if later than 0.5 s (lapse). If participants did not respond within 5 s, an alarm would sound to wake them up. The following performance outcome measures were evaluated: mean, median, and standard deviation of reaction times (RTs); mean RTs of the fastest 10% of trials and the slowest 10%; and the total number of lapses (RT > 0.5 s).

#### Lateralized attention task (LAT)

This was a 12-min visual-spatial reaction time task, modeled after the PVT. Six blocks (2 min each) alternated between having the left or right visual hemifield in white, and the other in black (Fig. 2*C*). Participants had to maintain fixation on a red rectangle in the center of the screen, and covertly attend to the white half of the screen. Every 2–10 s, a feint gray circle (1-cm radius, #F7F7F7) would appear randomly in any location of the illuminated hemifield and shrink away completely within 0.5 s. Participants were instructed to press a button before the circle disappeared, in which case the circle would freeze and flash green. Responses up to 0.5 s after the circle completely disappeared were considered late, no response within this time was a lapse, and a response at any other time a false alarm. If five stimuli were missed consecutively, an alarm would sound to wake up the participant. During the delay periods, 50-ms pink noise tones were presented every 1.5–5 s at ∼50 dB. Participants were instructed to ignore these tones. Performance outcome was measured as: mean, median, and standard deviation of RTs; mean RTs of the fastest 10% of trials and the slowest 10%; percentage of correct responses (0.1 s < RT < 0.5 s), late responses (0.5 s < RT < 1 s), and lapses (no response). Unlike the PVT, the LAT allows the distinction between slower RTs and complete lapses of attention.

#### Speech fluency task

Participants performed a tongue-twister reading task in English for 5–10 min. This consisted of 20 trials, one per sentence. Each sentence was repeated during each task block. A trial began with the sentence written on the screen (Fig. 2*D*). Participants were instructed to read it in their head once or twice to get familiar with it, but not practice speaking. When they were ready, they could press a button, and a green bar would appear below, steadily shrinking to count down a 10-s reading window. In this time, participants had to read out loud the sentence as many times as possible, as clearly as possible, and as correctly as possible, while their speech was being recorded. This was the only task in which the researcher was not in the room in order to reduce participants' self-consciousness. Performance outcome was measured as the number of correctly spoken words per second, and the number of mistaken words per second. Speech scoring was conducted manually by author SS, blinded to session and participant. A mistake was whenever a word was unfinished, not in the prompt, skipped, repeated (even partially, e.g., “se-seashells”), switched with a synonym (or any other unrelated word), or interrupted (e.g., by giggling). Switching two syllables of two words was counted as two mistakes (e.g., Yew Nork), whereas switching the order of two words was counted as one mistake.

#### Game

Participants played the mobile game BBTAN (based on the 1986 game *Arkanoid* by Taito) for 10 min (Fig. 2*E*). They started each session from level 1. The game involved a robot with a ball at the bottom of the screen, and a row of one to six bricks at the top. By tapping and dragging on the screen, participants could orient an arrow from the robot, and the ball would be launched from the robot in the indicated direction. The goal was to bounce the ball against the walls and hit as many bricks as possible, such that every time the ball hit a brick, the brick lost a point, and when the brick had no more points, it disappeared. At each round, after the ball was launched, hit the bricks, and bounced back to the bottom, the remaining set of bricks descended by one row, and a new row of bricks appeared at the top. When the bottom-most row of bricks reached the robot, the player lost the game. There were additional game features to help remove bricks faster. This was a “simple but addictive” game, requiring a minimum amount of spatial strategy to win, without any time pressure. No outcome measure was recorded for this task.

#### Music

Participants listened to two songs for 2.5 min each: the beginning of the instrumental soundtrack *Light of the Seven* composed by Ramin Djawadi from *Game of Thrones: Season 6*, and the beginning of the soundtrack *Finale (William Tell Overture)* composed by Hans Zimmer from *The Lone Ranger*.

### Questionnaires

A custom-built online survey tool, the Experiment Web Organizer for Questionnaires (EWOQ), was created for collecting questionnaire data through a web browser, written in React/typescript and hosted on Netlify and Google Cloud Platform. During the laboratory experiments, all questionnaires were filled out on a tablet, whereas the screening questionnaire and daily sleep reports were filled out on the participants' personal devices. Only the PSQI, MCTQ, and KSS are external, validated questionnaires. All others were created for this experiment and have not been tested on a broader population. The task questionnaires were conducted to evaluate subjective experiences during each task. Answers were given on a ∼10-cm continuous slider with labels, which are indicated on the y axes in Extended Data Figure 3-1.

### EEG recording and analysis

High-density EEG was recorded using HydroCel Geodesic Sensor Nets with 128 channels, connected to DC BrainAmp Amplifiers and recording software Brainvision Recorder (Vers. 1.23.0003, Brain Products GmbH). Data were recorded with a sampling rate of 1000 Hz with Cz reference. Impedances were set to be <5 kΩ for ground, reference, and external electrodes, and <25 kΩ for all other electrodes. After re-checking impedances, gel was refreshed every 4–6 h during the sleep deprivation bout, and in the morning after each night of sleep.

All data preprocessing, analysis, and statistics was done with custom scripts in MATLAB (R2019b) based on the EEGLAB toolbox v2019.1 (Delorme and Makeig, 2004). All further analyses involving source localization were performed with the FieldTrip toolbox v20210606 (Oostenveld et al., 2011).

#### Preprocessing

EEG data were filtered between 0.5 and 40 Hz and downsampled to 250 Hz. Visual detection of major artifacts and bad channels was conducted by author SS, blind to participant, task, and session. Overall, 4 ± 3 channels were removed on average per recording, out of 120 (Extended Data Fig. 6-2*A*). ICA was then used to remove physiological artifacts, mainly eye movements, heartbeat, and muscle activity (Dimigen, 2020). On average, 39 ± 12 components were removed from each recording (out of 106–122; Extended Data Fig. 6-2*B*). The Speech task had significantly more components removed, and the Music task the least. The majority of components removed were related to muscle artifacts. Bad channels were interpolated, and only the first 4 min of clean data were used, with average reference. The full pipeline is described in detail in Extended Data Figure 6-1.

#### Channel space power calculation

The power spectral density (PSD) estimate was calculated using MATLAB's *pwelch* function, with 8-s windows, Hanning tapered, and 75% overlap. To account for large interindividual differences in theta power (Extended Data Fig. 11-1) and the 1/f power amplitude distribution across frequencies, PSD for each frequency was z-scored. For theta topographies (e.g., Fig. 7), z-scored PSD values between 4 and 8 Hz were averaged. For power spectrums (Fig. 11), z-scored PSD values were averaged into three preselected regions of interest (ROIs): Front, Center, and Back. Exact channels are indicated in Extended Data Figure 5-1. For mean theta values (Fig. 5*B*), these ROI spectrum averages were further averaged between 4 and 8 Hz.

#### Source localization

Beamformer source localization was done with the dynamic imaging of coherent sources (DICS) algorithm from FieldTrip (Gross et al., 2001; Westner et al., 2022). A finite-element head model was implemented with the SimBio toolbox (Vorwerk et al., 2018) based on the segmentation of a standard MRI template brain. A 3D grid with 10-mm resolution (3294 voxels) was used as a source model. After being projected into the source space, power was z-scored for each frequency. For visualization, *t* tests were conducted for all gray-matter voxels, cluster corrected for multiple comparisons (Maris and Oostenveld, 2007; Maris, 2012), and significant clusters projected onto the inflated brain. To determine the main anatomic sources, z-scored data were parcellated based on the Automated Anatomical Labeling (AAL) atlas (Tzourio-Mazoyer et al., 2002). The median value of all voxels within each area was then averaged across frequencies. For both pipelines, only cortical areas were included, as there is currently little evidence that activity from deep brain structures reaches the scalp. The exact pipeline is provided in Extended Data Figure 8-1.

#### Trial analysis

Data from the STM task was separately analyzed by trial type, using data from the entire 25-min recording. Each trial was first divided into 2-s epochs for each window (encoding, first retention, second retention, and probe), and power was calculated with *pwelch* using a Hanning tapering window. The retention window was divided into two epochs to have the same duration as the encoding and probe epochs. Trials with >25% of samples marked as noise (during preprocessing step B in Extended Data Fig. 6-1) were excluded. The minimum number of trials for each memory load level for each session was 25. These remaining trials were then split by level and averaged. For each participant and each frequency, power values were then z-scored across epochs, trial types, channels, and sessions. The exact pipeline is provided in Extended Data Figure 5-1.

### Statistics

All parametric statistics were based on α = 5%. One PVT BL recording is missing, otherwise there were always 18 EEG datasets per task, per session.

#### ANOVAs

Each two-way repeated measures ANOVA (rmANOVA) was calculated using MATLAB's Statistics and Machine Learning Toolbox. Greenhouse–Geisser corrected *p*-values were always used because of occasional violations of sphericity; η^2^ effect sizes were calculated using the Measures of Effect Size (MES) Toolbox based on Hentschke and Stüttgen (2011).

#### *t* tests

whenever only two conditions were being compared, paired *t* tests were calculated. Hedge's *g* effect sizes are reported when *t* values are described in the text. These were calculated using the MES toolbox.

#### Correlations

Spearman's rank correlations were conducted between behavioral outcome measures and untransformed EEG theta power in preselected regions of interest. Untransformed power values were used to better capture interindividual differences.

#### False discovery rate (FDR) correction

Corrections for multiple comparisons were done by controlling for the false discovery rate, according to the procedure by Benjamini and Hochberg (1995). This was done using the Mass Univariate ERP Toolbox. FDR was chosen over other methods because it required the fewest a priori assumptions and thresholds (Groppe et al., 2011).

### Data and code availability

All data pertaining to this experiment (excluding personal identifiable information) will be shared by the corresponding authors on reasonable request. Original code is available on GitHub, including the data analysis (https://github.com/snipeso/Theta-SD-vs-WM), the STM task (https://github.com/snipeso/match2sample), the LAT (https://github.com/snipeso/LAT), the PVT (https://github.com/snipeso/pvt), and the Speech fluency task (https://github.com/snipeso/SFT).

## Results

### Changes in sleep architecture and subjective sleepiness confirm the effectiveness of the sleep deprivation protocol

To determine whether the sleep deprivation protocol was successful in increasing sleep pressure, we evaluated changes in sleep architecture between the baseline night and recovery night following sleep deprivation (Table 1). We found shorter sleep onset latencies (SOLs) and more deep sleep (NREM3), key indicators of increased sleep pressure.

**Table 1.** Sleep architecture

	Baseline	Prior night	Recovery night	Prior night vs baseline	Recovery night vs baseline	Recovery vs prior night
Wake	40.6 ± 30.1	18.1 ± 17.2	20.7 ± 9.3	0.001	0.008	0.544
NREM1	22.3 ± 12.3	10.8 ± 8.8	10.6 ± 4.8	<0.001	<0.001	0.919
NREM2	257.6 ± 34.2	115.1 ± 22.3	214.3 ± 51.6	<0.001	0.001	<0.001
NREM3	89.7 ± 40.1	64.6 ± 23.8	109.6 ± 37.0	<0.001	0.027	<0.001
REM	111.8 ± 32.0	33.4 ± 13.2	118.2 ± 29.8	<0.001	0.450	<0.001
SOL	16.8 ± 7.8	16.9 ± 13.2	5.6 ± 2.1	0.986	<0.001	0.002
SDu	481.8 ± 31.1	224.2 ± 16.8	453.0 ± 76.2	<0.001	0.108	<0.001
WASO	28.1 ± 26.0	5.7 ± 8.4	17.1 ± 9.0	0.001	0.076	0.001
SE (%)	92.5 ± 5.2	92.6 ± 7.0	95.6 ± 1.8	0.950	0.018	0.080
ROL	103.5 ± 47.5	98.7 ± 41.2	60.9 ± 19.4	0.458	<0.001	<0.001

All values in the first three columns are in mean minutes ± standard deviations, except SE, which is in percentages (100 × SDu/total time in bed). The last three columns indicate *p*-values from paired *t* tests between the different nights. *Prior night* refers to the 4-h night that begins the sleep deprivation session, and *recovery night* refers to the night after. REM, rapid eye movements (sleep); NREM, non-REM (sleep); SOL, sleep onset latency; SDu, sleep duration; WASO, wake after sleep onset; SE, sleep efficiency; ROL, REM onset latency.

All sleep stages except rapid eye movement sleep (REM) showed a significant change between baseline and recovery, with NREM3 increasing 30% at the expense of wake (−30%), NREM1 (−47%), and NREM2 (−16%). Sleep onset latency (SOL) significantly decreased from 16.8 to 5.6 min. Overall, sleep duration was shorter during the recovery night, although this was not statistically significant (*p* = 0.108), and sleep efficiency increased from 92% to 96%. Together, these results indicate that sleep pressure, specifically for slow wave sleep, increased over the 24-h wake period.

**Figure 3.**
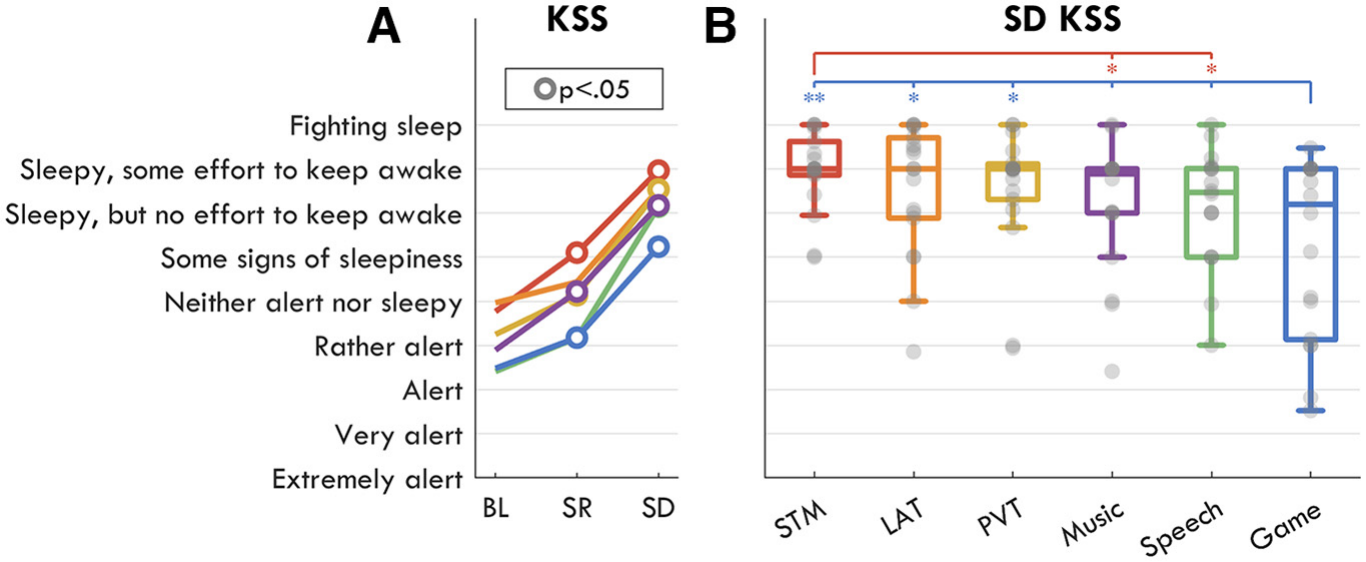
Subjective sleepiness ratings. Based on an adapted visual-analog Karolinska Sleepiness Scale (KSS) collected after each task. The labels were those of the original categorical KSS, but participants could choose intermediate values on the continuous scale. ***A***, Average scores for each task and each session. Each colored line represents a task (STM: red, LAT: orange, PVT: yellow, Speech: green, Game: blue, Music: purple). White-filled circles indicate a significant change from BL, FDR corrected for multiple comparisons. Extended Data Figure 3-1 provides the results for all other questions. ***B***, KSS scores during the SD task block. Gray circles represent each participant, the boxplot indicates median and interquartile range for each task. Stars indicate significant differences in paired *t* tests (the color indicates one task, the location of the star the other), FDR corrected for multiple comparisons, such that: *p < 0.05, **p < 0.01, ***p < 0.001. The empty tick mark indicates a trend (*p* < 0.1).

To determine the degree of sleep deprivation experienced by the participants, a two-way rmANOVA was conducted on KSS subjective sleepiness scores (Fig. 3*A*) with factors *session*, *task*, and their interaction (all other questionnaire data in Extended Data Fig. 3-1). There was a highly significant and very large effect of session (*F*_(2,30)_ = 35.42, *p* < 0.001, η^2^ = 0.355), a significant medium effect of task (*F*_(5,75)_ = 14.7, *p* < 0.001, η^2^ = 0.073) and a nonsignificant interaction (*F*_(10,150)_ = 0.96, *p* = 0.440, η^2^ = 0.008). This was the only subjective rating with a large effect of session, followed next by motivation (η^2^ = 0.07, all statistics in Extended Data Fig. 3-1). During sleep deprivation, participants felt less sleepy during the Game and most sleepy during the STM task (Fig. 3*B*).

10.1523/JNEUROSCI.1063-22.2022.f3-1 Extended Data Figure 3-1 Questionnaire answers for each task. All answers were given on a ∼10-cm slider with labels at specific intervals (indicated on the *y*-axis). White-filled circles indicate a significant change from BL, small colored circles a trend, FDR corrected. Each question was asked in this order. A two-way rmANOVA was conducted for each question with factors *session*, *task*, and their interaction. Due to occasional missing data, not all analyses included every participant, therefore for each statistic, the degrees of freedom (subscript in *F*_(A,B)_) indicate the sample size ($N=\frac{B}{A}+1$). No analysis had fewer than 16 participants, and the majority had all 18. ***A***, “Please indicate your sleepiness right now.” (Session: *F*_(2,30)_ = 35.42, *p* < 0.001, η^2^ = 0.35; Task: *F*_(5,75)_ = 14.7, *p* < 0.001, η^2^ = 0.073; Interaction: *F*_(10,150)_ = 0.96, *p* = 0.440, η^2^ = 0.008). ***B***, “How did you experience this task? [Relaxing].” (Session: *F*_(2,30)_ = 5.13, *p* = 0.012, η^2^ = 0.019; Task: *F*_(5,75)_ = 41.97, *p* < 0.001, η^2^ = 0.530; Interaction: *F*_(10,150)_ = 1.11, *p* = 0.365, η^2^ = 0.010). ***C***, “How did you experience this task? [Engaging].” (Session: *F*_(2,30)_ = 6.42, *p* = 0.015, η^2^ = 0.027; Task: *F*_(5,75)_ = 43.63, *p* < 0.001, η^2^ = 0.548; Interaction: *F*_(10,150)_ = 1.52, *p* = 0.191, η^2^ = 0.010). ***D***, “How focused on the task were you?” (Session: *F*_(2,30)_ = 5.25, *p* = 0.016, η^2^ = 0.039; Task: *F*_(5,75)_ = 13.63, *p* < 0.001, η^2^ = 0.235; Interaction: *F*_(10,150)_ = 0.39, *p* = 0.853, η^2^ = 0.006). ***E***, “How hard was it to perform this task?” (Session: *F*_(2,30)_ = 7.02, *p* = 0.006, η^2^ = 0.038; Task: *F*_(4,60)_ = 31.67, *p* < 0.001, η^2^ = 0.426; Interaction: *F*_(8,120)_ = 0.71, *p* = 0.585, η^2^ = 0.008). ***F***, “How much effort did you put into performing this task? (Think about how much you tried to do well, and how much more you could have done).” (Session: *F*_(2,28)_ = 2.17, *p* = 0.139, η^2^ = 0.022; Task: *F*_(4,56)_ = 4.84, *p* = 0.015, η^2^ = 0.114; Interaction: *F*_(8,120)_ = 0.37, *p* = 0.838, η^2^ = 0.006). ***G***, “How well do you think you did the task?” (Session: *F*_(2,30)_ = 2.02, *p* = 0.156, η^2^ = 0.025; Task: *F*_(4,60)_ = 8.96, *p* < 0.001, η^2^ = 0.134; Interaction: *F*_(8,120)_ = 1.04, *p* = 0.401, η^2^ = 0.023). ***H***, “How motivated were you during the task?” (Session: *F*_(2,20)_ = 6.93, *p* = 0.021, η^2^ = 0.070; Task: *F*_(5,50)_ = 19.61, *p* < 0.001, η^2^ = 0.381; Interaction: *F*_(10,100)_ = 3.33, *p* = 0.020, η^2^ = 0.04). Download Figure 3-1, TIF file.

### fmTheta is more localized than sdTheta

For fmTheta and sdTheta to be considered the same oscillation, they should originate from the same brain areas. To determine whether this was the case, we analyzed changes in theta from the short-term memory (STM) task during the retention window.

fmTheta was calculated by comparing z-scored power spectral density (PSD) changes between 4 and 8 Hz from L1 trials (one symbol to hold in memory) to L3 trials (three symbols to hold in memory), at BL during both the first and second retention epochs (Extended Data Fig. 4-1). Only the first epoch resulted in a significant increase in theta in any channel, therefore all further analyses were conducted on this epoch. L6 trials were also compared with L1 (Extended Data Fig. 4-1), but this did not yield different results from L3 versus L1. Because of the higher memory load, we had originally expected L6 to have more theta than L3. Given that performance for L6 trials was barely above chance level (Fig. 14*A*), we interpret this result as L6 being too difficult, causing participants to not engage in at least some of the trials. Therefore, we focused on L1 versus L3.

**Figure 4.**
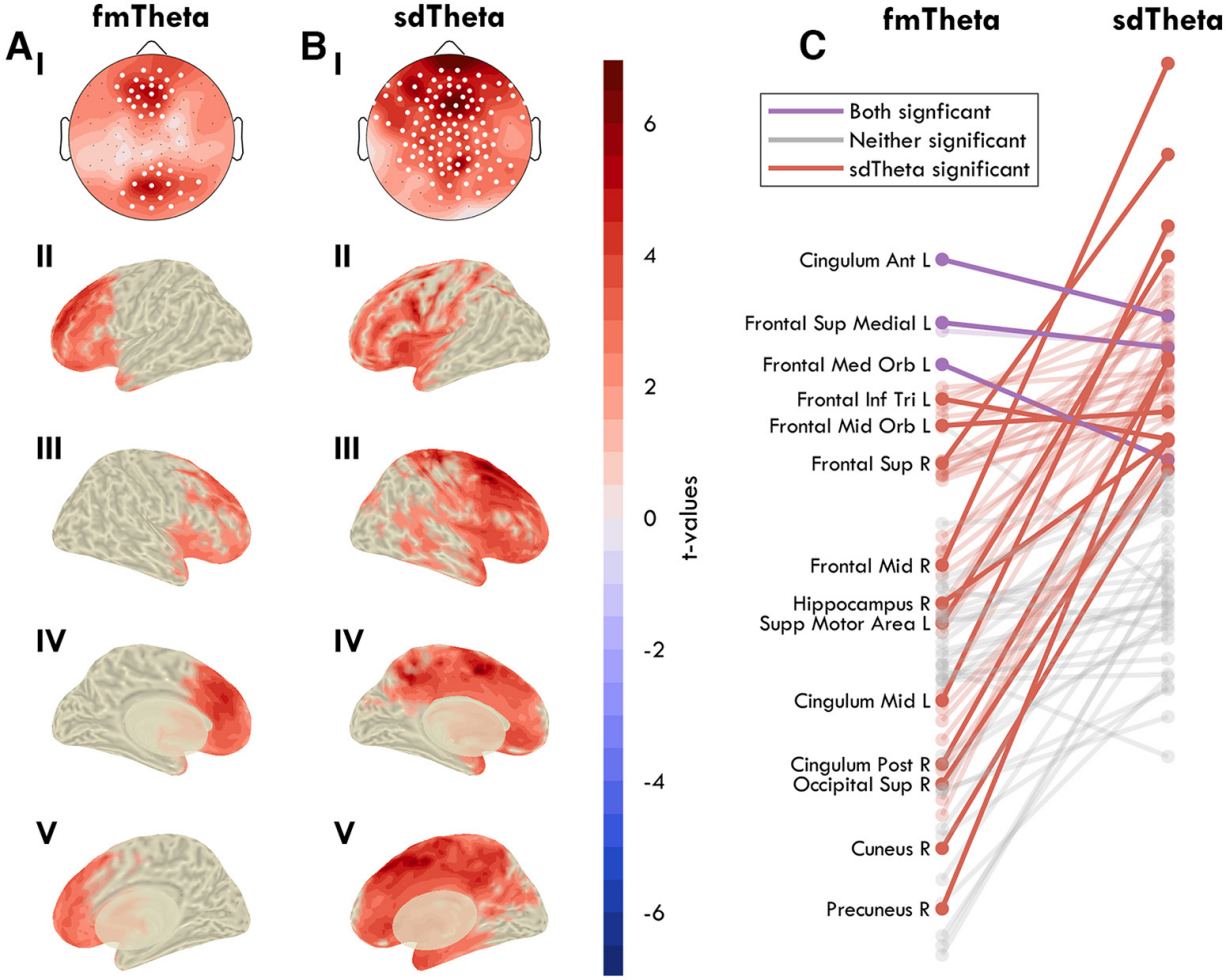
Sources of fmTheta and sdTheta. Theta is measured as average z-scored power between 4 and 8 Hz during the first retention epoch of the STM task. ***A***, Frontal-midline theta, calculated as the difference between trials with three items versus one item to hold in memory, from the BL session. ***B***, Sleep deprivation theta, calculated as the difference between SD trials and BL trials with one item to hold in memory. ***I***, Theta changes represented in a 2D topography of EEG channels, as a head seen from above (nose on top). Black dots indicate all channels, white dots indicate channels in which the change was statistically significant (*p* < 0.05) based on paired *t* tests, FDR corrected for multiple comparisons. Source localization is presented in ***II–V*** as inflated brains. *t* values are plotted with the same color scale in the channel and source space, such that red indicates a positive increase in theta from L1 to L3 in ***A*** and from BL to SD in ***B***. In the source space, voxel-wise cluster correction was implemented to mask nonsignificant effects. Extended Data Figure 4-1 provides the topographies also for the second retention window and L1 versus L6 trials. ***II***, Left hemisphere, lateral view. ***III***, Right hemisphere, lateral view. ***IV***, Left hemisphere, medial view. ***V***, Right hemisphere, medial view. ***C***, Change in *t* values for all areas between the fmTheta (***A***) and the sdTheta (***B***) comparisons, based on the AAL atlas. Lines in gray depict areas that showed no significant effects in either comparison, after FDR correction. Lines in red indicate areas showing a significant change in sdTheta, and lines in purple both in sdTheta and fmTheta. No area was only significant for fmTheta. Exact *t* values can be seen in Figure 9.

In the channel space, two significant channel groups emerged (Fig. 4*AI*): the frontal peaking over ch11 (Fz; *t*_(17)_ = 5.61, *p* = 0.002, Hedge's *g* = 0.76); the posterior peaking over ch75 (Oz; *t*_(17)_ = 5.61, *p* = 0.002, *g* = 0.76). Source localization identified the left medial frontal cortex as the main source (Fig. 4*AIV*), especially the anterior cingulate cortex (*t* = 4.76) and the superior frontal gyrus, medial (*t* = 4.06) as well as orbital part (*t* = 3.59; *t* values for anatomic areas provided in Fig. 9). These results replicate previous findings (Onton et al., 2005; Scheeringa et al., 2009; Michels et al., 2010; Ishii et al., 2014; Maurer et al., 2015). The right medial cortex also showed increases in theta; however, these areas did not survive correction for multiple comparisons.

10.1523/JNEUROSCI.1063-22.2022.f4-1 Extended Data Figure 4-1 Difference in theta power topographies between levels at BL for every epoch of the STM task. Level 1 is compared to level 3 (top row) and level 6 (bottom row) for each 2-s epoch. Encoding was when participants were viewing the items to memorize. Retention1 is the first half of the window in which participants had to hold the items in memory. Retention2 is the second half. Probe is when participants had to indicate whether a probe symbol was part of the original set. Participants' answers ended the probe window; therefore, this 2-s epoch could also encompass some of the rest window that followed. The color scale is the same as in Figure 4, with red indicating an increase in theta relative to L1. Download Figure 4-1, TIF file.

sdTheta was calculated using the same first retention epochs but comparing L1 trials from BL to L1 trials from SD (Fig. 4*B*). Unlike for fmTheta, this necessitates a between-session comparison. sdTheta was more widespread across the cortex than fmTheta, showing cluster-corrected increases in 38% of gray matter voxels relative to 21%, respectively. All areas showing load-effects of fmTheta were also significant for sdTheta (Fig. 4*C*), and the areas showing highest sdTheta were not among those significantly increasing in fmTheta. Specifically, the peak location of sdTheta was different in both the channel space (ch5) and source space: right middle frontal gyrus (*t* = 6.95) and superior frontal gyrus (*t* = 5.94; Fig. 4*BIII*). sdTheta extended along the medial cortex up to the cuneus (maximum *t* value *t*_max_ = 5.15) and was additionally present around the left insula (*t*_max_ = 4.58), and the temporal poles (*t*_max_ = 3.67). Therefore, sdTheta and fmTheta have different primary sources, and different spread throughout the cortex.

### fmTheta fades with increasing sleep deprivation

**Figure 5.**
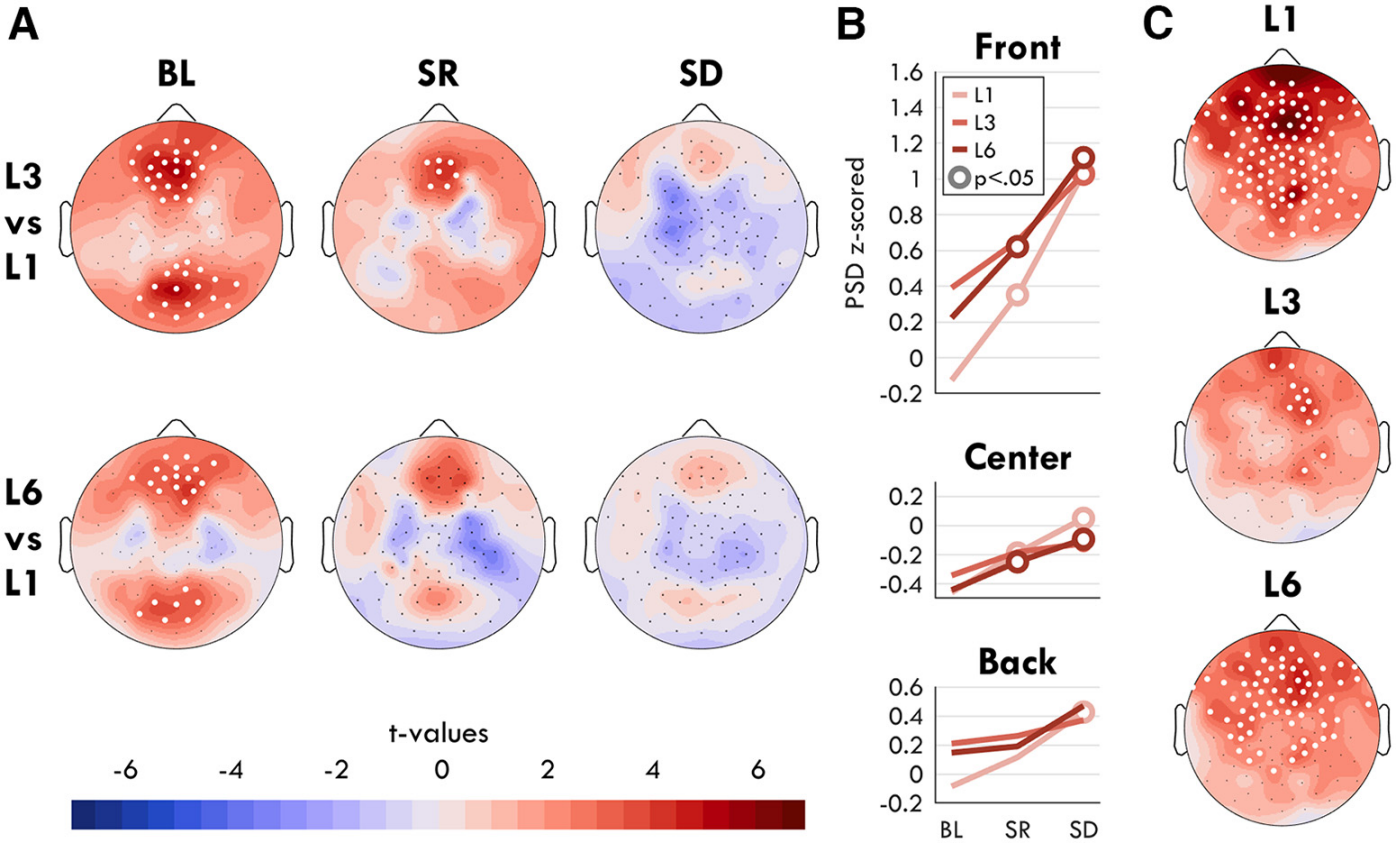
Interaction between STM task level and sleep deprivation theta power. ***A***, Difference in theta power for the first half of the retention period during the STM task between level 3 (top row) and level 6 (bottom row) relative to level 1 for every session. Color represents *t* values such that red indicates greater theta power in L3/L6 relative to L1. White dots indicate a significant effect, FDR corrected for multiple comparisons. ***B***, Mean z-scored theta power across sessions for each load level at each region of interest (ROI). White circles indicate a significant change from BL, filled circles a trend, FDR corrected for multiple comparisons. ***C***, Change in theta power topographies during the first retention epochs between SD and BL, split by memory load. Same color scale as ***A***, with red indicating more theta in SD relative to BL. Extended Data Figure 5-1 illustrates the analysis pipeline.

If sdTheta and fmTheta are independent oscillations, they should both be present during sleep deprivation when performing the STM task. fmTheta was therefore calculated at every session, for both L3 versus L1 and L6 versus L1 (Fig. 5*A*). Surprisingly, fmTheta decreased in amplitude with increasing sleep deprivation, until no channel showed statistically significant differences with memory load during SD.

10.1523/JNEUROSCI.1063-22.2022.f5-1 Extended Data Figure 5-1 STM EEG analysis pipeline. Starting data was preprocessed as described in Extended Data Figure 6-1. Sections in black indicate steps in common between more than one analysis. Purple indicates steps for calculating power spectrums, teal indicates steps specific to topographies, and green steps for average region of interest (ROI) power. The ROI channels were preselected, and in the diagram the Front ROI is in blue (3, 4, 5, 6, 9, 10, 11, 12, 13, 15, 16, 18, 19, 20, 22, 23, 24, 112, 118, 124), Center in yellow (7, 30, 31, 35, 36, 37, 41, 42, 47, 51, 52, 53, 54, 55, 60, 61, 62, 78, 79, 80, 85, 86, 87, 92, 93, 97, 98, 103, 104, 105, 106, 110, 129), and Back in red (65, 66, 69, 70, 71, 74, 75, 76, 82, 83, 84, 89, 90). STM epochs were all of 2 s in duration; however, the encoding epoch was shifted 0.1 s earlier to avoid initial retention EEG responses. The pipeline for all other task EEG analyses (Fig. 7) is identical, except without trials or epoching, and using 8-s windows with 75% overlap across the first 4 min of data. Download Figure 5-1, TIF file.

A two-way rmANOVA was conducted with factors *session*, *load,* and their interaction, separately for three regions of interest (ROIs). In the Front ROI there was both a significant and large effect of session (*F*_(2,34)_ = 17.17, *p* < 0.001, η^2^ = 0.287), a significant but small effect of load (*F*_(2,34)_ = 5.92, *p* = 0.008, η^2^ = 0.030), and a small significant interaction (*F*_(4,68)_ = 3.74, *p* = 0.017, η^2^ = 0.016). In the Center ROI there was a significant effect of session (*F*_(2,34)_ = 10.16, *p* < 0.001, η^2^ = 0.198), no effect of load (*F*_(2,34)_ = 1.35, *p* = 0.271, η^2^ = 0.006), and a trending interaction (*F*_(4,68)_ = 2.37, *p* = 0.095, η^2^ = 0.022). In the Back ROI there was a significant effect of session (*F*_(2,34)_ = 4.64, *p* = 0.028, η^2^ = 0.072), a small trending effect of load (*F*_(2,34)_ = 2.63, *p* = 0.096, η^2^ = 0.013), and a significant interaction (*F*_(4,68)_ = 3.88, *p* = 0.019, η^2^ = 0.014).

The interaction between load and session was driven by a larger increase in theta for low memory load trials during sleep deprivation (Fig. 5*B*). To better understand this, we compared sdTheta topographies (BL vs SD) for each memory load level (Fig. 5*C*). L1 showed the largest and most widespread increase in theta (*t*_max_ = 7.28, *p* < 0.001, *g* = 1.57), L3 the lowest and most local increase (*t*_max_ = 4.32, *p* = 0.024, *g* = 0.87), and L6 was intermediate (*t*_max_ = 4.93, *p* = 0.007, *g* = 1.45). As a result of sdTheta increasing more in low memory load trials, fmTheta effectively disappeared. However, given that for most of the participants these three sessions were performed in order, it is also possible that this disappearing fmTheta is driven by a repetition effect, although previous studies (Habeck et al., 2004) have not found repetition effects of behavior in this task design.

### Sources of sdTheta are task dependent

**Figure 6.**
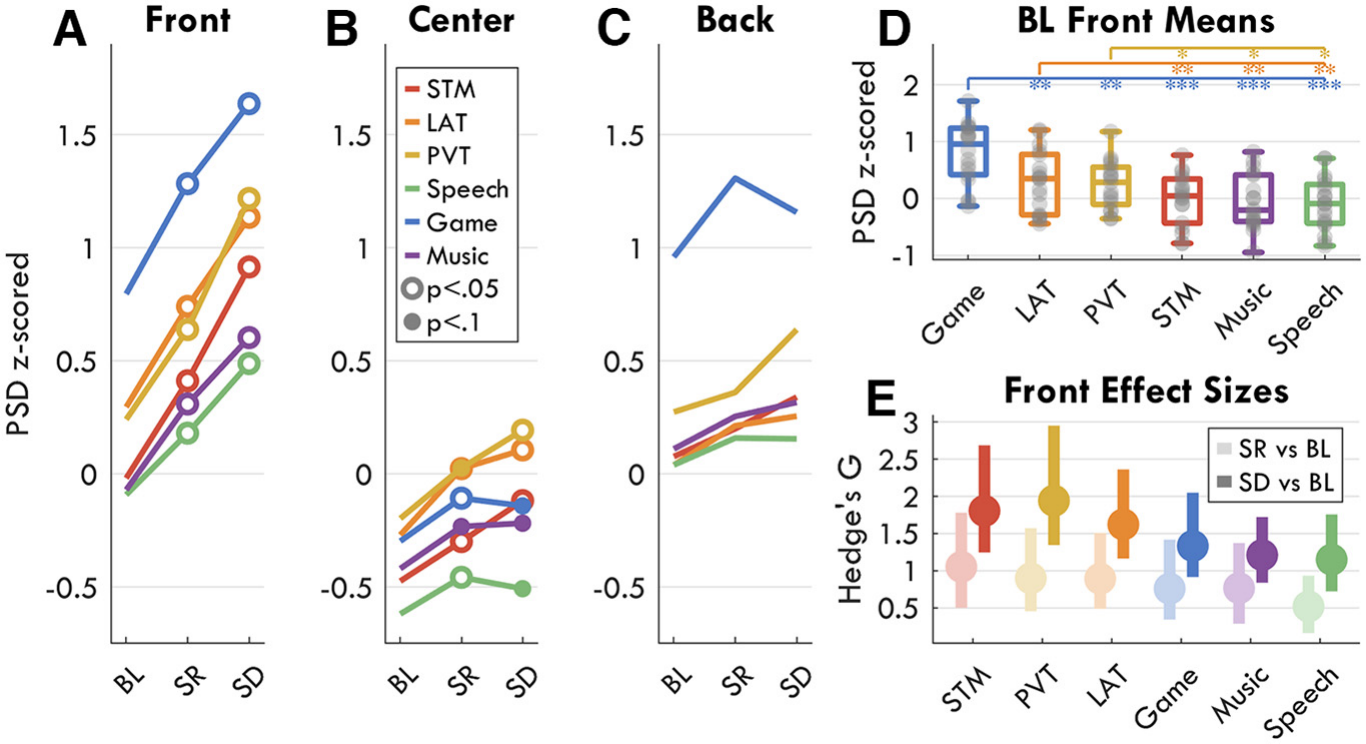
Change in theta across sessions for all tasks by region of interest (ROI). Mean z-scored theta power for three ROIs: front (***A***), center (***B***), and back (***C***). Open circles indicate within each task a significant change in theta relative to BL, filled circles indicate a trend, based on paired *t* tests, FDR corrected for multiple comparisons within each plot. ***D***, Mean theta power for all tasks at baseline in the front ROI. Gray circles represent each participant, the boxplot indicates median and interquartile range. Stars indicate significant differences between tasks (the color indicates one task, the location of the stars the other) such that: *p < 0.05, **p < 0.01, ***p < 0.001. ***E***, Hedge's *g* effect sizes of the changes in theta in the front ROI from BL to SR (light colors) and SD (dark colors). The disk indicates Hedge's *g*, the bars indicate 95% confidence intervals. Extended Data Figure 6-1 illustrates the preprocessing pipeline. Extended Data Figure 6-2 indicates the channels and components removed during the preprocessing.

**Figure 7.**
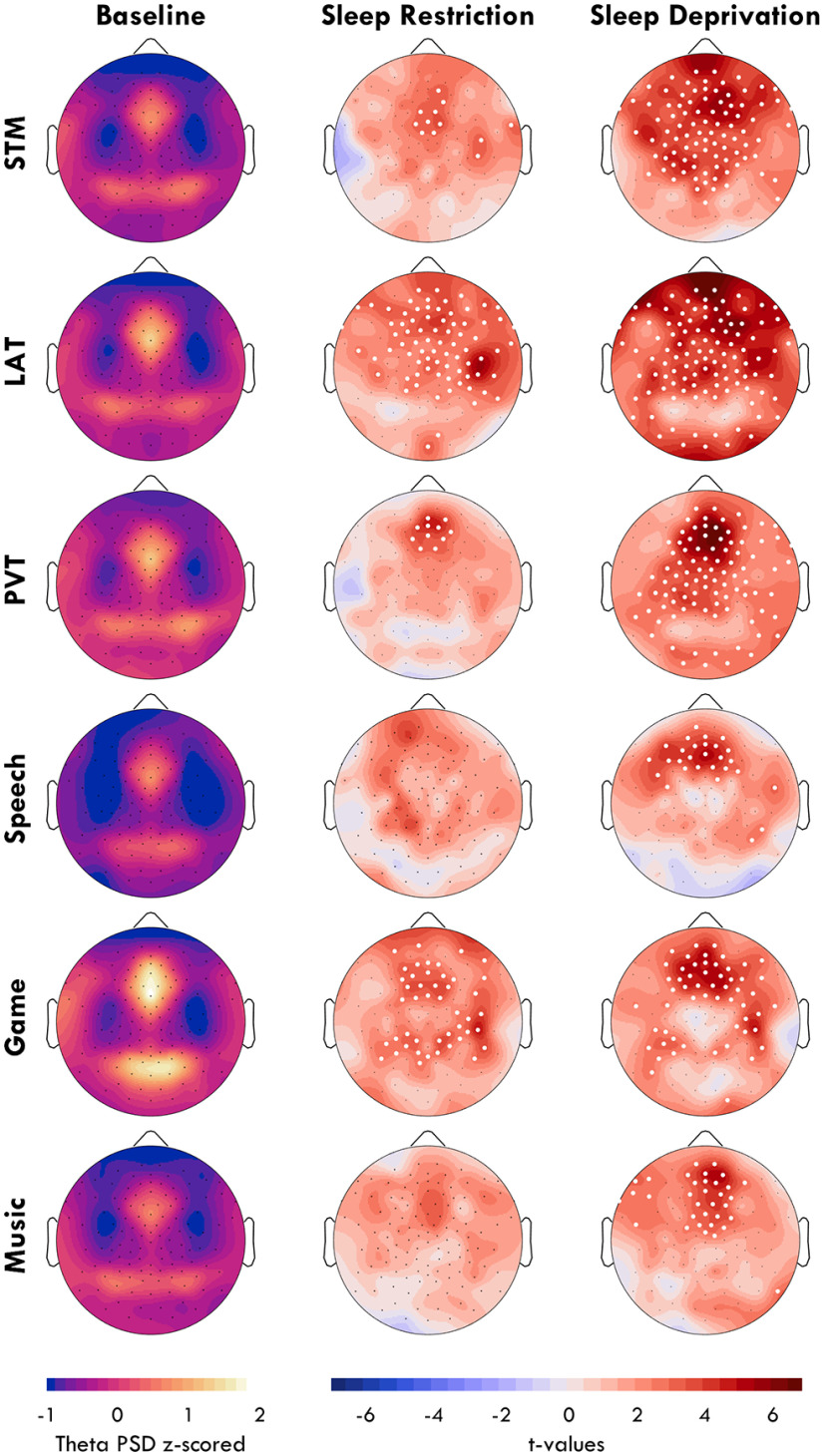
Theta sleep deprivation topographies by task. First column: mean z-scored theta power topographies at BL. Second and third column: the change in theta power from BL to SR and SD, respectively. Color indicates *t* values, with red indicating an increase relative to BL. Black dots indicate all channels, white dots indicate channels in which the change was statistically significant (*p* < 0.05), FDR corrected for multiple comparisons.

**Figure 8.**
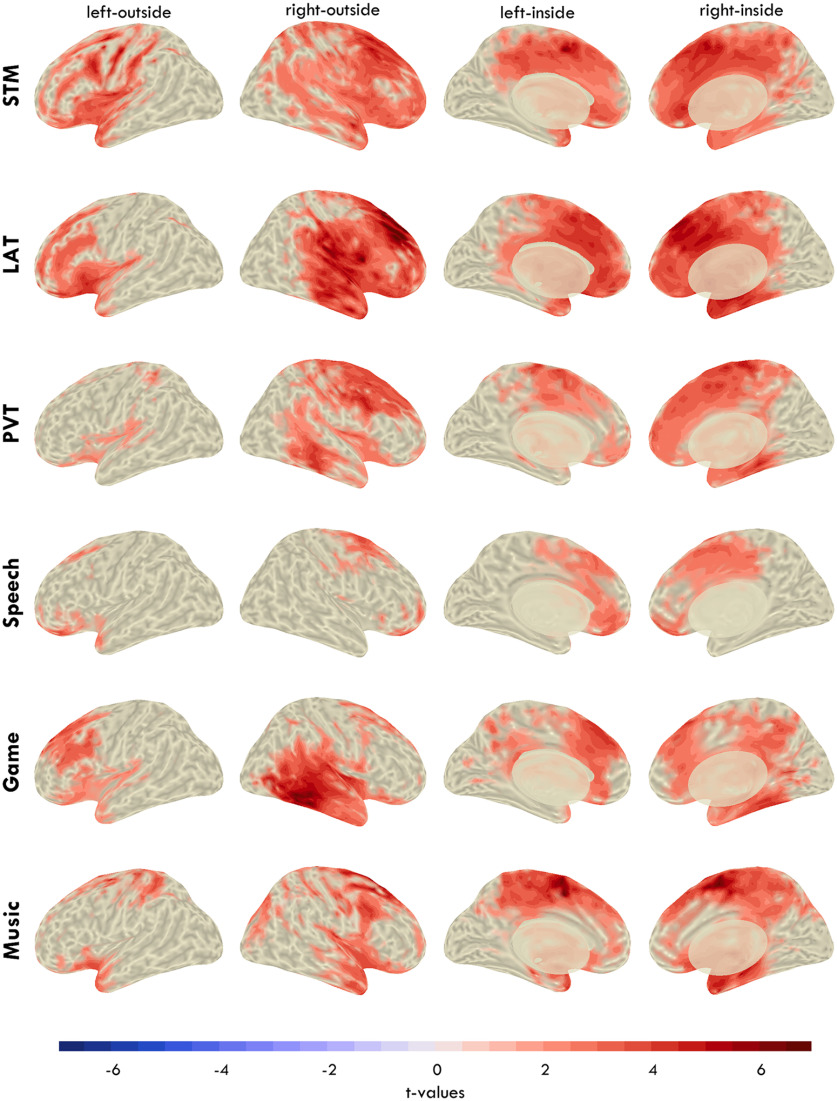
Change in theta from BL to SD in the source space projected on inflated brains. Color indicates *t* values, such that red indicates an increase in power from BL to SD. Voxel-wise cluster correction was implemented to mask nonsignificant effects. Extended Data Figure 8-1 illustrates the analysis pipeline for the source localization.

**Figure 9.**
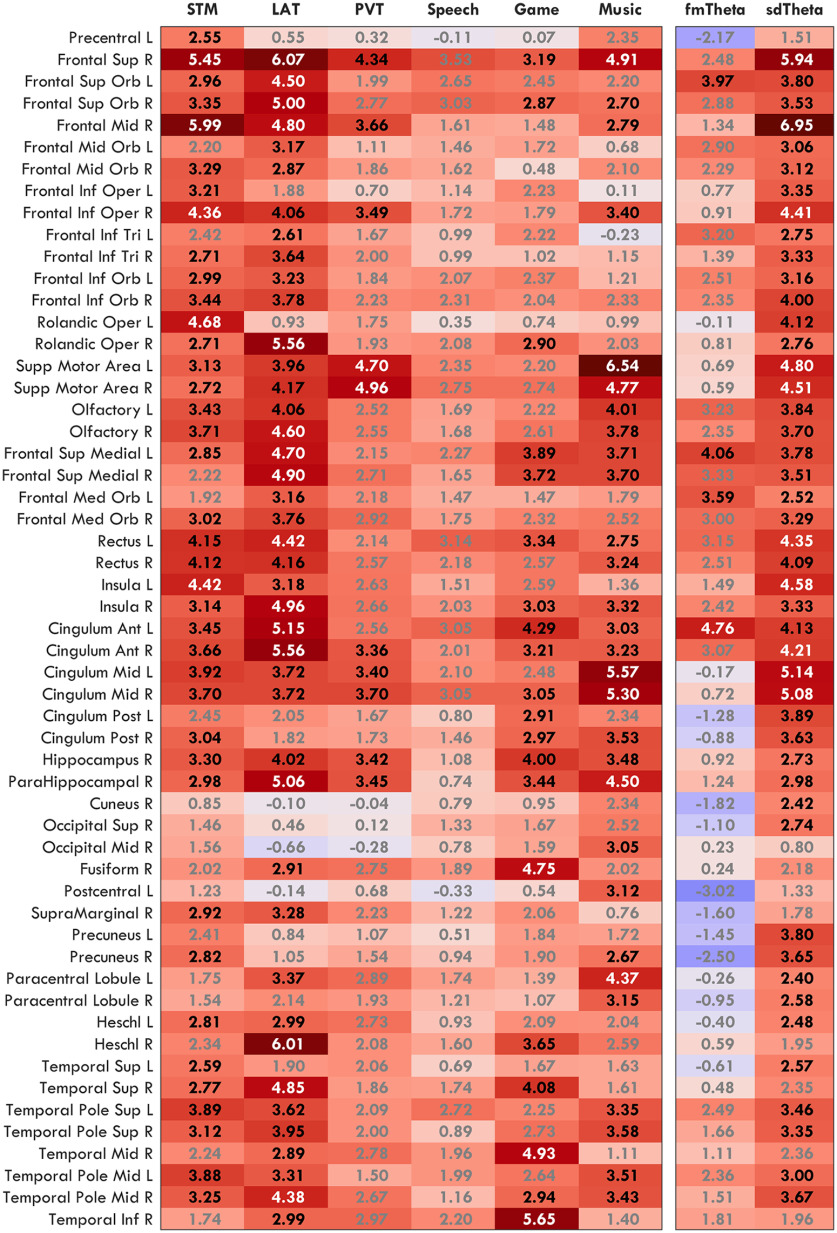
*t* values of the change in theta by anatomic source. Only areas with at least one significant test (BL vs SD in all tasks; L3 vs L1 from fmTheta and L1 BL vs L1 SD for sdTheta in the STM) are included. Text in gray indicates areas not significant after FDR correcting for multiple comparisons. Text in white indicates the top 10% of *t* values in the whole table.

The results from Figure 4 show distinct topographies for fmTheta and sdTheta. The literature has identified fmTheta to consistently originate from the same medial region; however, similar source localization has never been done for sdTheta. To determine whether sdTheta is consistent or task dependent, we compared theta changes from BL in 6 different tasks. Mean theta values for all tasks in regions of interest are provided in Figure 6. Figure 7 depicts the sdTheta changes for both SR and SD relative to BL in the channel space, and Figure 8 provides the source localization for SD relative to BL displayed on inflated brains. Figure 9 provides the *t* values for all anatomic regions found to be significant in at least one comparison of SD relative to BL.

10.1523/JNEUROSCI.1063-22.2022.f6-1 Extended Data Figure 6-1 Pipeline for data preprocessing. Red steps were conducted on the data used for the power analysis (250-Hz sampling rate, 0.5–40 Hz). Blue steps were conducted on the data used for the independent component analysis (ICA; 500-Hz sampling rate, 2.5–100 Hz). White-filled steps (***B***, ***G***, ***I***) involved manual work. ***A***, First, data was low-pass filtered at 40 Hz using EEGLAB's default filter. A Kaiser notch filter was then applied to remove 50-Hz line noise and subsequent harmonics. Data was then down-sampled to 250 Hz. A 0.5-Hz high-pass Kaiser-window-based FIR filter was then applied (0.25-Hz stopband, 60-dB stopband attenuation, 0.05 passband ripple). ***B***, The data were visually inspected to identify bad channels (Extended Data Fig. 6-2*A*), bad time windows, and bad single-channel segments (i.e., snippets). Bad channels were considered as such that if they contained any nonphysiological signals (anything not from the brain, muscles, or eyes) that occurred either continuously or repeatedly throughout the recording. Furthermore, external channels outside the EGI net were automatically removed (49, 56, 104, 113), as well as the face channels (126, 127). Bad time windows were any segments in time in which an artifact affected multiple channels at once, often due to body movements or brief muscle clenching. Bad snippets were nonphysiological artifacts affecting only a few channels. ***C***, Prior to removing artifacts with ICA, bad channels were removed, snippets interpolated, and the data re-referenced to the average. However, bad time windows were not removed. These are removed later (***J***). ***D***, Data used for calculating the ICA were filtered and downsampled differently from ***A*** to maximize the detection of eye-movement artifacts. ***E***, ***F***, For ICA, only clean data were used, Cz was restored, and all channels re-referenced to the average. EEGLAB's “runica” ICA algorithm was applied, with principal component analysis (PCA) rank reduction. Using EEGLAB's ICLabel function (v1.2.4), the first 60 components were automatically classified as either brain data or artifacts. Components were marked for removal if they had a “brain” classification value lower than 0.1 but restored if they had a classification as “other” larger than 0.6 (i.e., an unknown component). ***G***, Visual inspection was then conducted to correct any misclassifications, or mark for removal additional bad components outside the first 60 (total components removed in Extended Data Fig. 6-2*B*). ***H***, ***I***, After the components were removed, one final visual inspection of the data was done to remove any additional bad channels, and possibly repeat the preprocessing if notable artifacts remained in the data. ***J***, To have the same amount of data for each task, the first 4 min of clean data were used to calculate power. ***K***, Channels 17, 48, and 119 were further removed from all datasets, as these were often removed due to poor signal quality in steps ***B*** and ***I***. Channel 129 was removed for the source localization as its coordinates were not available. Download Figure 6-1, TIF file.

10.1523/JNEUROSCI.1063-22.2022.f6-2 Extended Data Figure 6-2 Removed channels and components for each recording and each session. ***A***, Number of channels removed (out of 120). Each colored line represents a participant. The black line is the group average. ***B***, Number of removed components after ICA (max 120). Asterisks indicate significant differences from paired *t* tests between sessions, FDR corrected, such that *p < 0.05, **p < 0.01, ***p < 0.001. Download Figure 6-2, TIF file.

10.1523/JNEUROSCI.1063-22.2022.f8-1 Extended Data Figure 8-1 Pipeline for source localization analysis. ***A***, To compute theta power for the source localization, we used a fast Fourier transform (FFT) with a Hanning taper, applied to each 8-s window. To construct the forward model, we obtained a finite-element head model, implemented with the SimBio toolbox based on the segmentation of the template T1 MRI image from the Montreal Neurological Institute into gray matter, white matter, cerebrospinal fluid, scalp, and skull. Subsequently, a standard 3D grid (10-mm spacing, 3294 voxels inside the head) and the head model were used to compute the leadfield matrix. To avoid depth bias, the leadfield was normalized. As an inverse solution, we used the DICS beamformer technique. We first computed common spatial filters based on a cross-spectral density matrix obtained from pooled conditions, with a regularization parameter lambda set to 5%. The precomputed common spatial filters were then applied independently to each condition. After projecting each recording to the source space, each frequency was z-scored for each participant (as done in the channel space). ***B***, 3D brain source maps. Nonparametric cluster correction was implemented instead of FDR because it was only intended as a mask for the inflated brains plots, and not as hypothesis testing. First, independent samples *t* tests for all voxels were done for the contrast of interest (two-tailed, *p* < 0.05). Next, significant neighboring voxels were clustered if they showed the same direction of effect. To assess the statistical significance of each cluster, a cluster-level test statistic was calculated by computing the sum of all *t* values in the cluster. The significance of each cluster was estimated by comparing the cluster-level test statistic to a reference permutation distribution derived from the data. The reference distribution was obtained by randomly permuting the data 5000 times. The cluster *p*-value was estimated as the proportion of the elements in the reference distribution exceeding the cluster-level test statistic. For the visual representation of results, significant clusters of *t* values were projected on the inflated brain surface. Due to uncertainty regarding the ability of surface EEG to detect deep brain structures' electrophysiological activity (thalamus, amygdala, etc.), these areas were covered in a patch and not included in the next analysis. ***C***, We additionally performed parcellation of the grid into 80 regions of interest (parcels), in accordance with the AAL atlas. Regions of the basal ganglia and cerebellum were excluded from further analysis. The median power for each frequency across voxels was used for each anatomical area. Power for all theta frequencies was then averaged, and paired *t* tests were conducted for each parcel, FDR correcting for multiple comparisons. FDR, false discovery rate; AAL, automated anatomical labeling atlas; DICS, dynamic imaging of coherent sources; MRI, magnetic resonance imaging; MNI, Montreal Neurological Institute; FFT, fast Fourier transform. Download Figure 8-1, TIF file.

A two-way rmANOVA was conducted for each ROI with factors *session*, *task*, and their interaction (mean values in Fig. 6*A–C*). The Front ROI had a significant effect of session (*F*_(2,32)_ = 28.02, *p* < 0.001, η^2^ = 0.224), a significant effect of task (*F*_(5,80)_ = 22.51, *p* < 0.001, η^2^ = 0.249), and a significant interaction (*F*_(2,160)_ = 1.88, *p* = 0.090, η^2^ = 0.010). The Center ROI also had a significant effect of session (*F*_(2,32)_ = 13.09, *p* < 0.001, η^2^ = 0.105), a significant effect of task (*F*_(5,80)_ = 14.05, *p* < 0.001, η^2^ = 0.239), and a significant interaction (*F*_(2,160)_ = 2.53, *p* = 0.035, η^2^ = 0.021). The Back ROI did not have a significant effect of session (*F*_(2,32)_ = 2.41, *p* = 0.111, η^2^ = 0.021), but a strong effect of task (*F*_(5,80)_ = 21.67, *p* < 0.001, η^2^ = 0.305), and no interaction (*F*_(2,160)_ = 0.79, *p* = 0.549, η^2^ = 0.007). Therefore, although the effects were small, sdTheta was significantly task dependent. While the Game had the overall highest frontal theta (Fig. 6*D*), the increase with sleep deprivation was more pronounced in the STM, PVT, and LAT (Fig. 6*E*).

When comparing theta changes across the whole topography, all tasks showed increases in theta between BL and SR in most channels; however, no channel was significant for the Speech and Music conditions after FDR correction (Fig. 7, center). The highest overall increase was seen for the LAT over ch109 (*t*_max_ = 5.74, *p* = 0.002, *g* = 1.33), accompanied by widespread increases. Because of the otherwise medium-low effect sizes, the comparison between BL and SR was not further investigated with source localization. However, these results demonstrate already in the channel space how task-specific changes are present also when controlling for circadian time.

From BL to SD, the task-specific sdTheta topographies become even more evident (Fig. 7, right). The LAT, STM, and PVT showed the most widespread increases, as well as the highest amplitude (PVT: *t*_max_ = 7.52, *p* < 0.001, *g* = 1.85; LAT: *t*_max_ = 7.10, *p* < 0.001, *g* = 1.24; STM: *t*_max_ = 6.31, *p* = 0.001, *g* = 1.80). The Speech task showed the lowest and most local increase in theta (*t*_max_ = 5.50, *p* = 0.005, *g* = 1.51).

The source space allowed further anatomic localization of the origin of theta. All tasks except the Game showed a predominantly right, frontal increase in theta (Fig. 8), although no anatomic area survived FDR correction for the Speech task (Fig. 9). One of the primary sources of sdTheta across all tasks was the right superior frontal gyrus. All tasks (except Speech) also had significant theta originating from the right hippocampus, parahippocampus, anterior and middle cingulate cortex. The STM and LAT had further extensive increases across both dorsal and medial frontal areas, with the STM showing high theta activity along the left lateral sulcus (Rolandic operculum, insula), and the LAT in the right lateral sulcus (Heschl's gyrus, rolandic operculum, insula). Unfortunately, source localization along this sulcus is challenging because of how gray matter is folded and may require subject-specific MRI structural scans for accurate results.

The overall strongest source of sdTheta was the left supplementary motor area during the Music task (Fig. 9, *t*_L_ = 6.54), extending contralaterally (*t*_R_ = 4.77) as well as into the middle cingulate cortex. Bilateral supplementary motor areas were also the main sources of theta for the PVT (*t*_L_ = 4.70, *t*_R_ = 4.96). The supplementary motor area showed significant increases in the LAT and STM but to a lesser extent (STM: *t*_L_ = 3.13; LAT: *t*_R_ = 4.17) and were not significant in the Game.

Finally, the most atypical distribution of sdTheta came from the Game (Fig. 8), which showed minimal increases in frontal cortices and primary sdTheta originating from the right inferior temporal cortex (inferior temporal gyrus, mid temporal gyrus, fusiform gyrus; *t*_max_ = 5.65). The only other task to show significant sdTheta in these regions, to a lesser extent, was the LAT (inferior temporal gyrus, *t* = 2.99).

Overall, the majority of sdTheta occurred in medial and superior frontal cortices, with a right lateralization. LAT and STM were the most widespread in the source space (Fig. 8; 39% and 35% of significant voxels, respectively), the Game, Music, and PVT intermediate (28%, 27%, 25%), and Speech the least (9%). While most sdTheta sources were frontal, there were substantial differences between tasks. The high theta from the supplementary motor area in the Music task and in the inferior temporal cortex in the Game suggests a preference of sdTheta for cortical areas not critical for the ongoing behavioral task.

### sdTheta power spectrums have multiple peaks

Figure 7, left column, illustrates how the average theta power at baseline more resembles fmTheta (Fig. 4*AI*), especially for the Game, than it does sdTheta within tasks. This suggests that sdTheta occurs in addition to task-related fmTheta found at BL. In order to determine whether sdTheta could be further distinguished from this baseline fmTheta, we inspected the spectrograms of the different tasks for all participants. In particular, we were interested in whether tasks with high frontal BL theta showed an additional distinct peak in the theta range following sleep deprivation. This would support the hypothesis of theta during sleep deprivation as a separate oscillation from task-related, baseline fmTheta.

**Figure 10.**
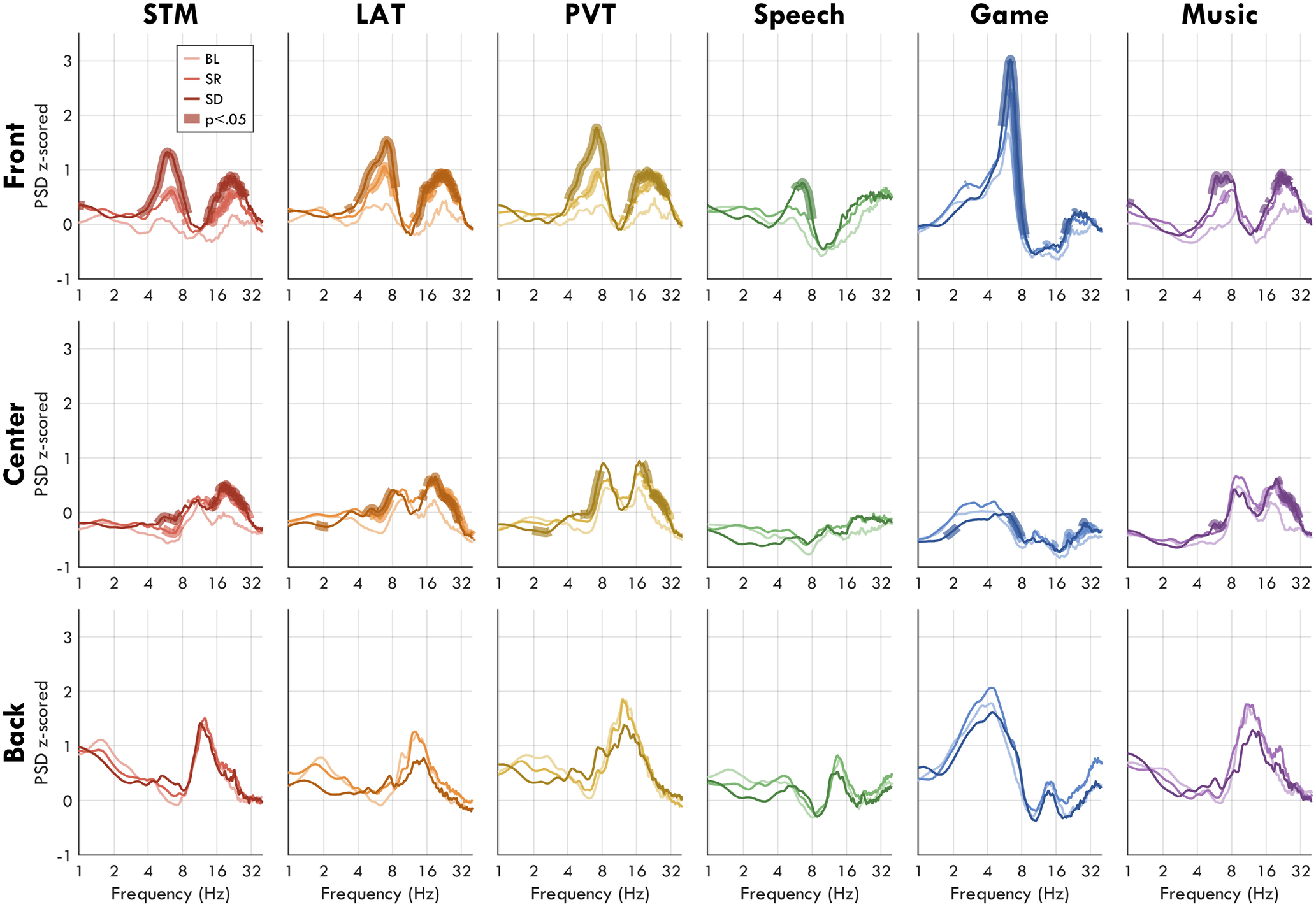
Average z-scored power spectrums from each ROI for each task. Thin lines indicate the spectrum at each session, averaged across participants. Thick lines indicate statistically significant changes (paired *t* tests, *p* < 0.05, FDR corrected) for a given frequency relative to BL. The frequency axis is log-transformed. The *y*-axis represents power spectral density, z-scored. Note, while there is an increase of both theta and beta (15–25 Hz) with sleep deprivation, the lack of increase in the delta (1–4 Hz) and alpha (8–12 Hz) ranges indicate that the spectral changes are not because of broadband increases.

**Figure 11.**
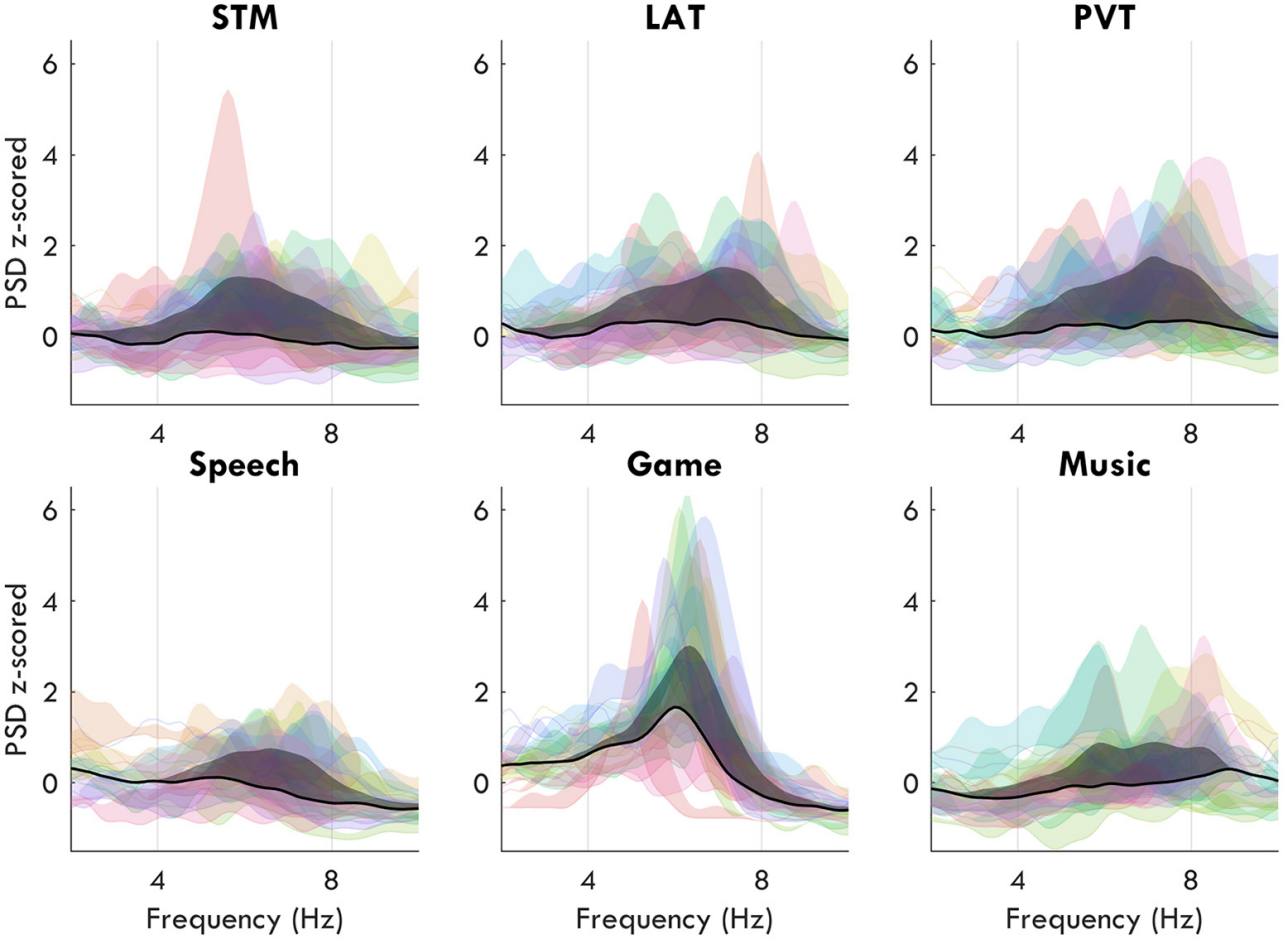
Z-scored power spectrums from the front ROI for each task. Overlapping spectrums from the front ROI of each task for every participant. The base curve of each colored patch represents the BL spectrum, the upper curve the SD spectrum, and the filled-in area reflects the increase in power. The average power change across participants is the final patch in black. Extended Data Figure 11-1 provides the uncorrected spectrums.

**Figure 12.**
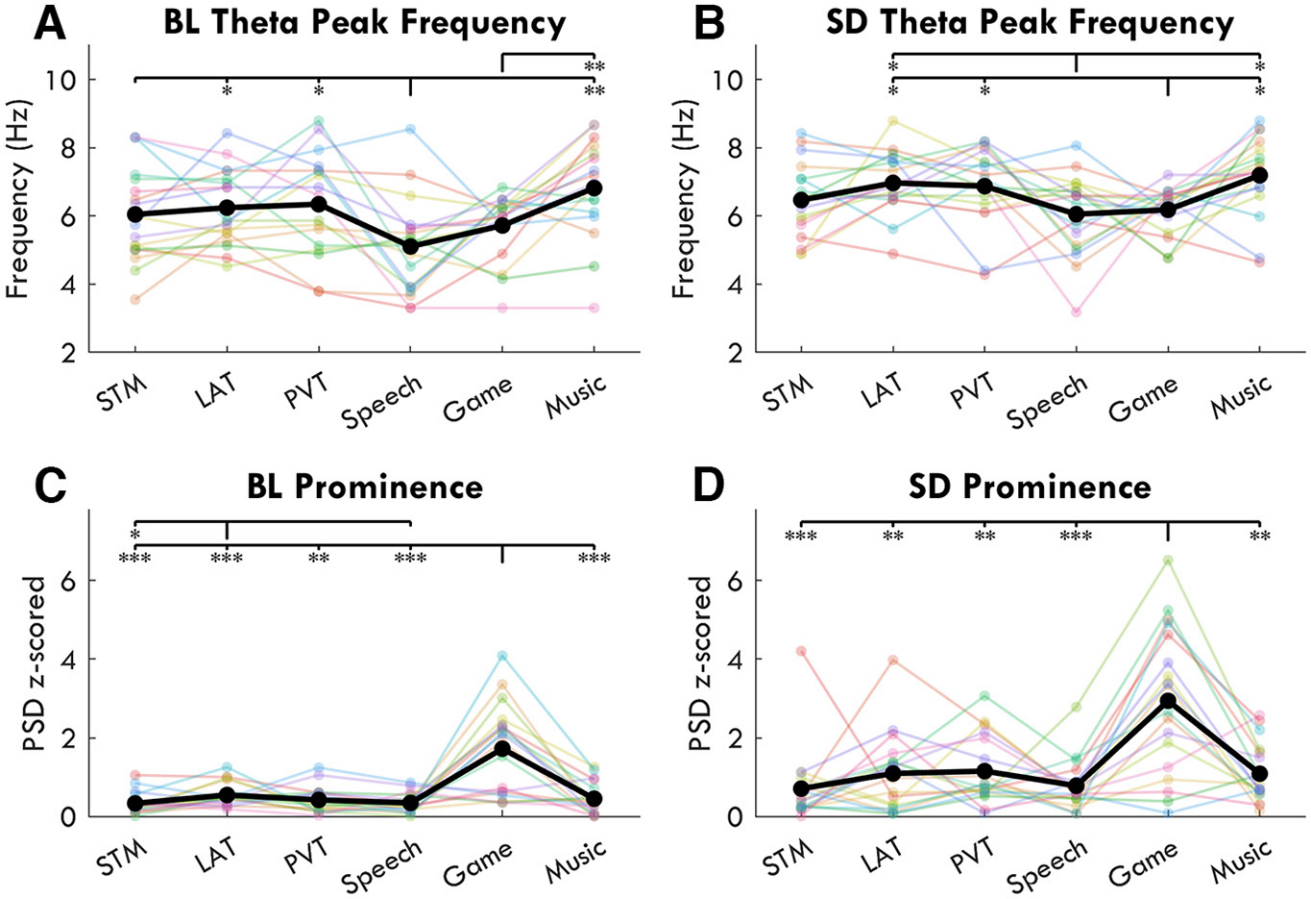
Prominence and peak frequency of z-scored power spectrums from the Front ROI. Each color represents a different participant, the black line indicates the average. Asterisks indicate significant differences from paired *t* tests between tasks, FDR corrected, such that: *p < 0.05, **p < 0.01, ***p < 0.001. ***A***, ***B***, The highest amplitude peak in the 3- to 9-Hz range. ***C***, ***D***, Prominence refers to the amplitude difference between the highest peak and the closest trough to that peak within a 3- to 9-Hz range.

Paired *t* tests between BL and SR/SD z-scored power spectrums confirmed that the effect of sleep deprivation was specific to the theta range, resulting in a prominent peak in the average SD Front ROI spectrum for all tasks (Fig. 10). However, when inspecting individual participants' spectrums, sdTheta often did not occupy a single consistent peak within or across individuals (Fig. 11). Instead, individuals' peaks were spread over the entire theta range, often with multiple smaller peaks within the same participant. Furthermore, the maximum peak frequency for a given participant was not consistent across tasks (Fig. 12*A*,*B*).

10.1523/JNEUROSCI.1063-22.2022.f11-1 Extended Data Figure 11-1 Uncorrected power spectrums from the front ROI for each task. Overlapping EEG power spectrums, untransformed, from the Front ROI of each task for every participant. The base curve of each colored patch represents the BL spectrum, the upper curve represents the SD spectrum, and the filled-in area reflects the increase in power. The average power change is the final patch in black. Download Figure 11-1, TIF file.

The exception was the Game, which showed the overall highest amplitude frontal theta as well as the most clearly defined peak both during BL and SD (Fig. 12*C*,*D*), with prominence values (calculated as the difference in *z*-scores between the maximum theta amplitude and the closest trough in the spectrum) of 1.72 ± 1.14 (mean ± SD) at BL and 2.94 ± 1.88 at SD. By contrast, the STM task had a prominence of 0.33 ± 0.27 at BL, and 0.71 ± 0.94 at SD. Unexpectedly, the STM task had low BL frontal theta, similar to Speech and Music (Fig. 6*D*).

Because of the clear presence of fmTheta at BL in the Game, we considered this task to be the most likely to show both an fmTheta peak and an sdTheta peak during SD. The BL peak frequency was significantly different from the SD peak frequency, increasing from 5.7 ± 1.0 Hz to 6.4 ± 0.5 Hz (*t*_(17)_ = 2.62, *p* = 0.018, *g* = 0.84). For reference, the STM peak was 6.0 ± 1.4 Hz at BL, and 6.4 ± 0.7 Hz at SD, but the increase was not statistically significant (*t*_(17)_ = 0.87, *p* = 0.397, *g* = 0.30). However, as can be seen in the individual Game spectrums in Figure 11, only a single peak is present for most participants, with the baseline theta peak merely shifted in frequency and increased in amplitude during SD. Multiple peaks were instead found in all other tasks during SD, which may indicate a multitude of different theta oscillations not found in the Game.

**Figure 13.**
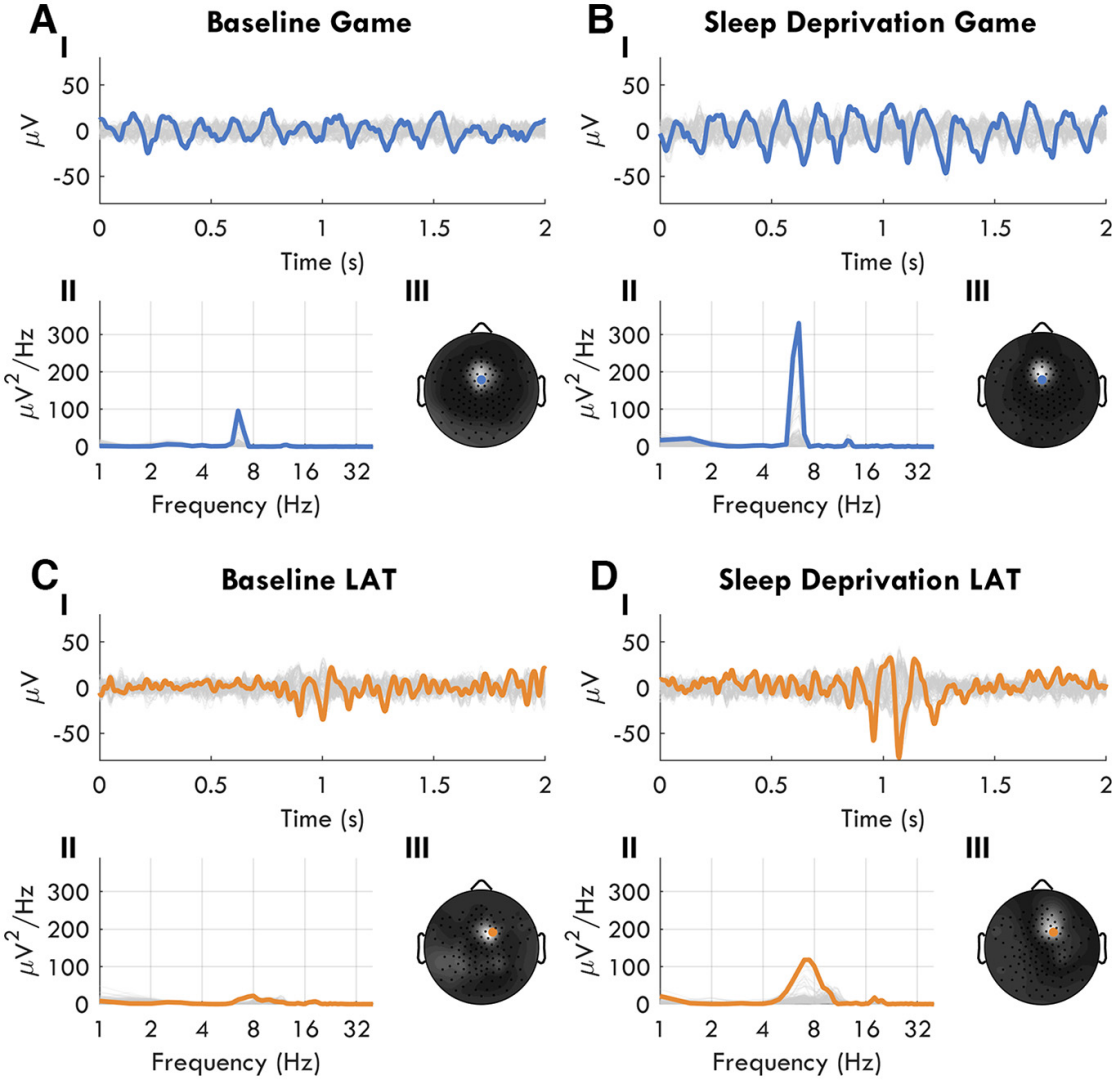
Examples of theta bursts. Taken from the same participant during BL (***A***, ***C***) and SD (***B***, ***D***), and from the Game (***A***, ***B***) and the LAT (***C***, ***D***). ***I***, EEG data in time, amplitude in microvolts. All channels are represented in gray, and the channel expressing the highest theta in color. ***II***, Power spectrums of all channels in gray, and peak theta channel in color. The frequency axis is log-transformed. ***III***, Average theta power mapped across all channels from the 2 s shown in ***I***. The scale is normalized for each plot separately to the min-max. Colored dot indicates the same channel highlighted in ***I*** and ***II*** (ch6 for Game, ch118 for LAT).

Visual inspection of the EEG data provided further insight into task-related theta differences. At BL, fmTheta bursts as described by Mitchell et al. (2008) were visible primarily in the Game task (Fig. 13*A*) in 11 individuals. These were frontal midline bursts that lasted 1–5 s with amplitudes around 15–20 μV. No other types of prominent theta oscillations were similarly detectable by visual inspection in any task at BL (best example, Fig. 13*C*). During SD, fmTheta became even more prominent in the Game EEG (Fig. 13*B*), with higher amplitudes and longer bursts, appearing for 13 participants and increasing in other tasks as well. In addition to fmTheta, widespread bursts often with frontal peaks appeared during sleep deprivation especially in the LAT and STM (Fig. 13*D*). These had a much shorter duration (two to three oscillations), but with a higher peak amplitude (>40 μV). As can be seen from the spectrums (Fig. 13*II*), Game theta bursts yielded narrow-band theta power, whereas the LAT bursts had more widespread spectrums. These examples support an interpretation of at least two types of oscillations in the theta range that increase with sleep deprivation.

### Short-term memory performance does not relate to either fmTheta or sdTheta

Given that the presence of sdTheta and fmTheta are dependent on the ongoing task, we wished to explore whether there was a relationship between theta and behavioral outcomes. If fmTheta is functionally relevant, or if sdTheta is a form of local sleep, then the changes in theta across individuals should correlate with the extent of behavioral deficits.

Maurer et al. (2015) found that the increase in fmTheta with short-term memory load was negatively correlated with the change in accuracy, such that the more fmTheta, the worse participants performed with increasing load. We did not replicate this correlation for either the first retention epoch (*r*_(16)_ = –0.05, *p* = 0.850) nor the second (*r*_(16)_ = –0.30, *p* = 0.233).

**Figure 14.**
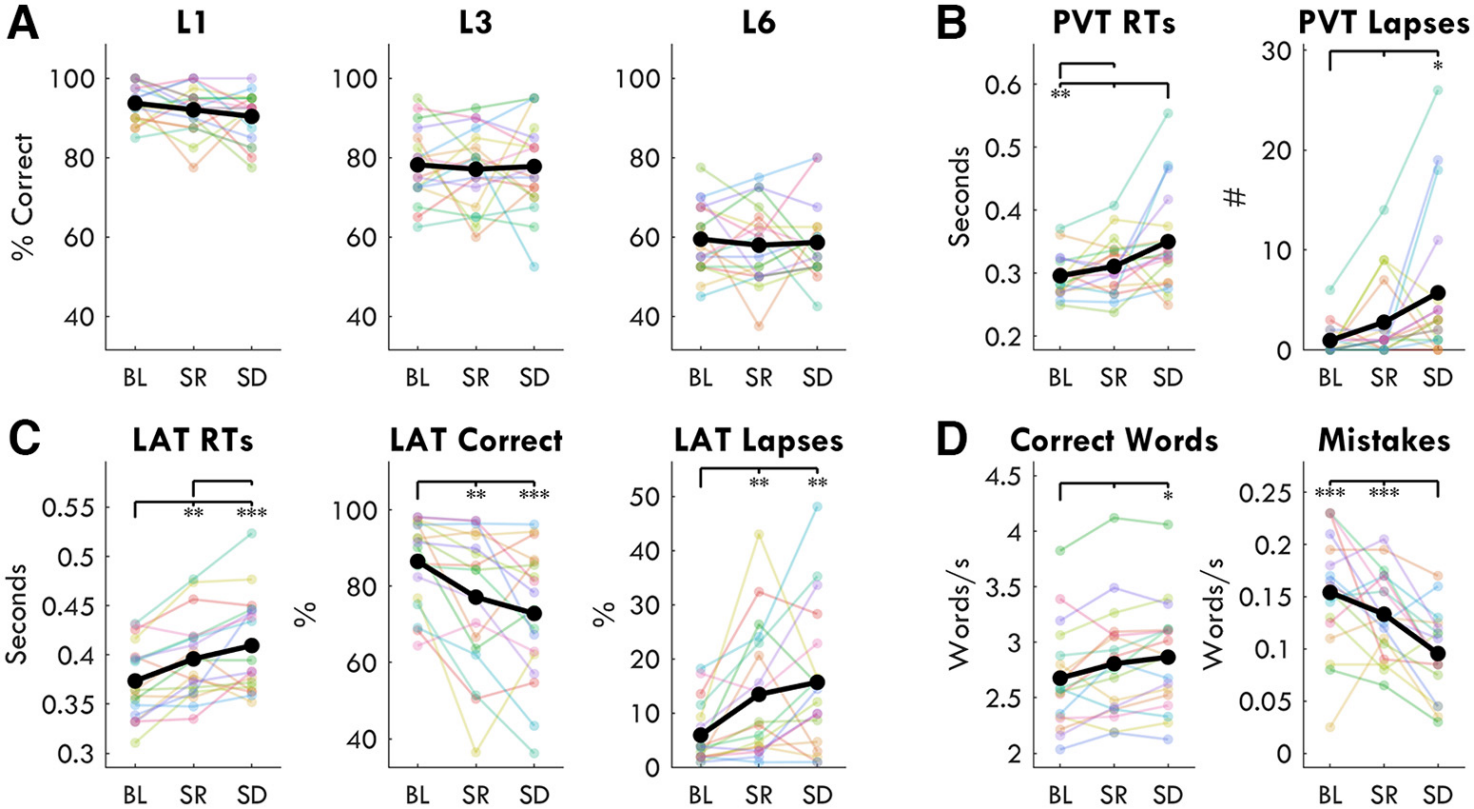
Task performance. ***A***, STM recall accuracy for every memory load level (1, 3, 6) at every session. The *y*-axis indicates percentage of correctly identified probes (both true positives and correct rejections). Thin lines indicate individual participants, thick lines indicate the mean. Chance level was 50%. No level showed a significant change from BL. ***B***, PVT performance. Left: mean reaction times (RT) in seconds. Right: number of trials for which the RT > 0.5 s. ***C***, LAT performance. Left: mean RTs. Middle: percentage of trials for which the RT was between 0.1 and 0.5 s (i.e., while the stimulus was still visible). Right: percentage of trials for which no response was given. ***D***, Speech fluency task performance. Left: rate of correct words per second across sessions. Right: rate of mistaken words per second across sessions. Asterisks indicate significant differences from paired *t* tests between sessions, FDR corrected, such that: *p < 0.05, **p < 0.01, ***p < 0.001. Extended Data Figure 14-1 highlights the performance for the four participants who conducted the baseline after the sleep deprivation bout.

Before determining whether there was any correlation between STM performance and sdTheta, we evaluated whether there was an effect of sleep deprivation on performance using a two-way rmANOVA with factors *session*, *level*, and their interaction. We found no effect of session (*F*_(2,34)_ = 0.45, *p* = 0.636, η^2^ = 0.002), a very large effect of level (*F*_(2,34)_ = 275.68, *p* < 0.001, η^2^ = 0.717), and no significant interaction (*F*_(4,68)_ = 0.43, *p* = 0.717, η^2^ = 0.001). Performance accuracy across sessions is provided in Figure 14*A*.

10.1523/JNEUROSCI.1063-22.2022.f14-1 Extended Data Figure 14-1 Task performance by experiment order. Same data as Figure 14. Participants who conducted the baseline night after the sleep deprivation session are highlighted in blue (*N* = 4), the remainder are in yellow (*N* = 14). Download Figure 14-1, TIF file.

Despite the lack of an effect of sleep deprivation on STM accuracy, we still performed correlations between the change in performance for each memory load level and the change in theta power from BL to SD for the three ROIs. Neither the Front (L1: *r*_(16)_ = –0.04, *p* = 0.862; L3: *r*_(16)_ = –0.02, *p* = 0.935; L6: *r*_(16)_ = –0.13, *p* = 0.605), Center (L1: *r*_(16)_ = –0.06, *p* = 0.810; L3: *r*_(16)_ = –0.14, *p* = 0.585; L6 *r*_(16)_ = –0.08, *p* = 0.749) nor Back ROI (L1: *r*_(16)_ = –0.15, *p* = 0.542; L3: *r*_(16)_ = –0.37, *p* = 0.128; L6: *r*_(16)_ = –0.30, *p* = 0.231) showed significant correlations between the difference in theta and the difference in behavior. Therefore, short-term memory performance accuracy was not related to either fmTheta or sdTheta.

### Behavioral performance is not directly related to the increase in sdTheta

**Figure 15.**
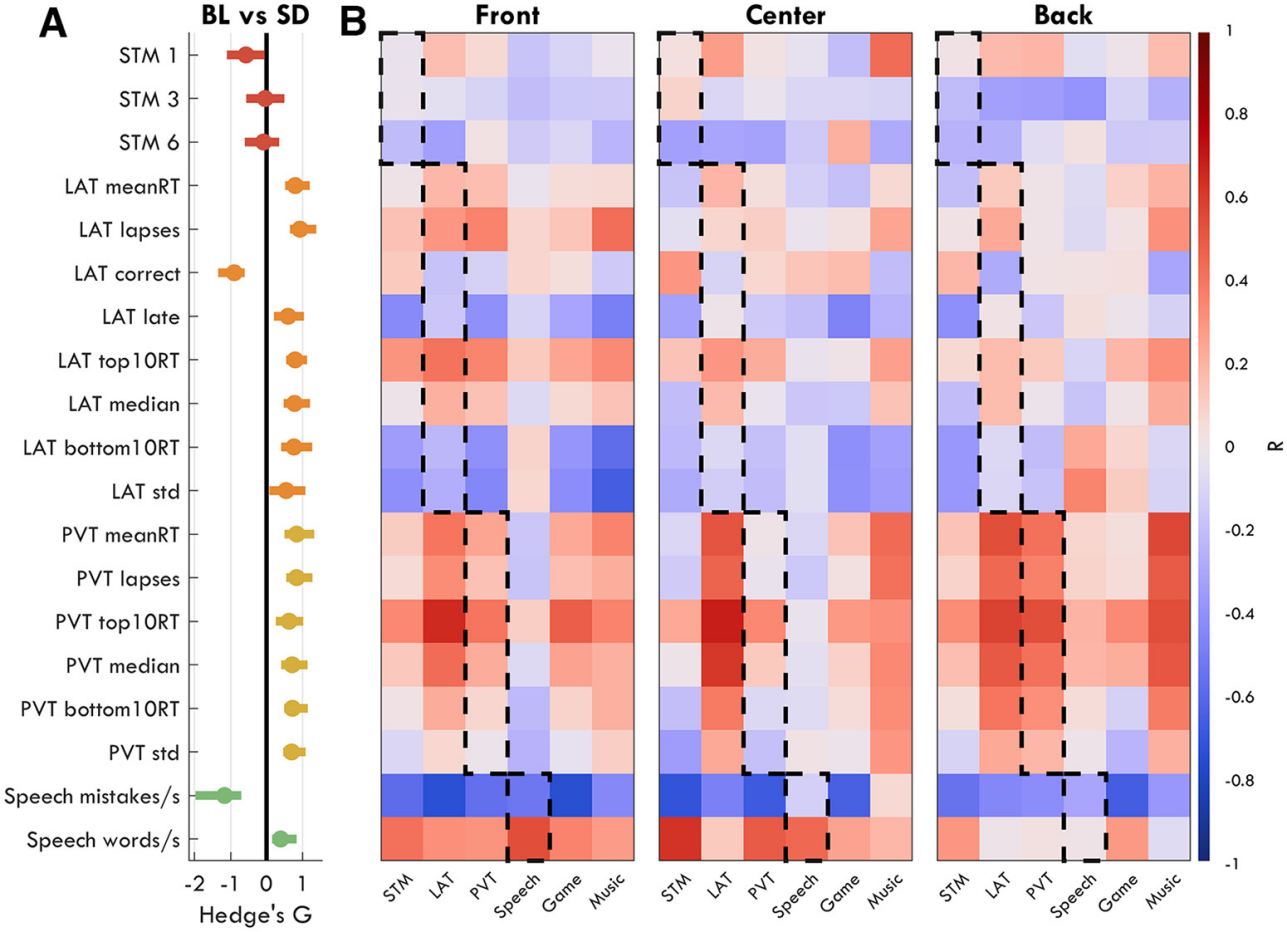
Correlations between changes in behavioral performance and changes in theta for each ROI. ***A***, Hedge's *g* effect sizes for paired *t* tests comparing BL to SD for each behavioral outcome measure. Bars indicate 95% confidence intervals. Positive values indicate an increase in that outcome measure from BL to SD. ***B***, *R* values for each pairwise correlation between behavioral measure and theta power for each task for each ROI. Comparisons within the same task are outlined with a dotted edge. Red indicates positive correlations, blue indicates negative. Note, the *R* values within the dotted line are not higher than outside it.

In rats, local sleep events were found to result in behavioral lapses in a reaching task (Vyazovskiy et al., 2011). Therefore, we expected that an increase in response lapses in the PVT and LAT would correlate with increases in theta. More generally, to determine whether the occurrence of sdTheta could affect any behavioral measure, we first established which outcome measures changed significantly with sleep deprivation (Figs. 14, 15*A*), and then correlated the change from BL to SD for each performance measure with the change in theta from BL to SD for each ROI (Fig. 15*B*).

STM performance accuracy for all three memory load levels were the only measures which did not show a statistically significant change with sleep deprivation (as anticipated by the previously described two-way ANOVA). The PVT (Fig. 14*B*) and LAT (Fig. 14*C*) showed a worsening of performance with increased reaction times (PVT: *t*_(16)_ = −3.45, *p* = 0.003, *g* = 0.84; LAT: *t*_(17)_ = −4.51, *p* < 0.001, *g* = 0.80) and increased number of lapses (PVT: *t*_(16)_ = −2.94, *p* = 0.010, *g* = 0.84; LAT: *t*_(17)_ = −4.44, *p* < 0.001, *g* = 0.93), consistent with the literature (Basner and Dinges, 2011). The Speech task (Fig. 14*D*) unexpectedly showed a significant reduction in the number of mistakes (*t*_(17)_ = 4.81, *p* < 0.001, *g* = −1.17) and an increase in words per minute (*t*_(17)_ = −3.16, *p* = 0.006, *g* = 0.39). N.B. these two variables were not significantly correlated between each other (*r*_(16)_ = –0.37, *p* = 0.129) although they both showed improvement with sleep deprivation.

The PVT has previously been shown to be unaffected by task repetition (Basner et al., 2018), and the outcome measures of both the PVT and LAT performed under soporific conditions in this experiment all returned to baseline following recovery sleep (data not shown). Similarly, the STM task has also been shown to be unaffected by task repetition (Habeck et al., 2004), albeit with two repetitions instead of three. Therefore, the behavioral changes in the Speech task are the only ones that may have been affected by learning.

To determine whether any of these behavioral changes in performance (both positive and negative) were related to sdTheta, we correlated each measure with the change in untransformed theta power for each ROI during the first 4 min of the respective tasks. A significant correlation was found between the decrease in number of Speech mistakes per minute and the increase in frontal theta (*r*_(16)_ = –0.53, *p* = 0.025), as well as number of correct words per minute (*r*_(16)_ = 0.54, *p* = 0.023), such that the more participants improved, the more theta they had. The increase in mean reaction times (RTs) of the fastest 10% of responses of the PVT was positively correlated with the increase in theta over the Back ROI (*r*_(15)_ = 0.54, *p* = 0.027). No other performance measure showed a significant correlation with sdTheta of the same task.

In a previous sleep deprivation study, Gorgoni et al. (2014) also found a positive correlation between the increase in the mean of the fastest 10% of PVT RTs with increases in centro-posterior theta power. However, theta was measured during a separate resting EEG, recorded just before the task. Inspired by this, we then correlated all of our behavioral outcome measures with sdTheta in *all* tasks, for each ROI (Fig. 15*B*). No correlation between behavioral outcome measure and sdTheta survived FDR correction for multiple comparisons, including the within-task ones previously described. We therefore provide the correlations with uncorrected *p*-values and limit ourselves to cautious interpretations.

Similar to Gorgoni and colleagues, for the PVT we found significant positive correlations for the increase in fastest 10% RTs with the LAT sdTheta (Front: *r*_(15)_ = 0.65, *p* = 0.006, Center: *r*_(15)_ = 0.69, *p* = 0.003, Back: *r*_(15)_ = 0.58, *p* = 0.016) and Music sdTheta (Back: *r*_(15)_ = 0.55, *p* = 0.025). Significant correlations were additionally found with the LAT sdTheta and mean PVT RTs (Center: *r*_(15)_ = 0.52, *p* = 0.034, Back: *r*_(15)_ = 0.54, *p* = 0.027) and median RTs (Center: *r*_(15)_ = 0.61, *p* = 0.010, Back: *r*_(15)_ = 0.50, *p* = 0.041), as well as with Back Music sdTheta and mean RTs (*r*_(15)_ = 0.57, *p* = 0.019), median RTs (*r*_(15)_ = 0.51, *p* = 0.038), and lapses (Back: *r*_(15)_ = 0.50, *p* = 0.043).

Notably, despite robust decreases in LAT performance with sleep deprivation (Fig. 15*A*), no outcome measure was significantly correlated with LAT sdTheta. Instead, significant negative correlations were found between Front Music and LAT late responses (*r*_(16)_ = –0.49, *p* = 0.041), the slowest 10% of RTs (*r*_(16)_ = –0.57, *p* = 0.015), and the standard deviation of RTs (*r*_(16)_ = –0.66, *p* = 0.004).

Finally, the reduction in mistakes in the Speech task was significantly negatively correlated with Front sdTheta in all tasks except trending in Music (STM: *r*_(16)_ = –0.58, *p* = 0.012; LAT: *r*_(16)_ = –0.73, *p* = 0.001; PVT: *r*_(15)_ = –0.57, *p* = 0.017, Speech: *r*_(16)_ = –0.53, *p* = 0.025; Game: *r*_(16)_ = –0.74, *p* < 0.001; Music: *r*_(16)_ = –0.44, *p* = 0.067), and to a lesser extent significant in the Center ROI for sdTheta in the STM, LAT, and Game, and Back sdTheta in the STM and Game. Notably, the correlations between mistakes and theta were higher for the STM, PVT, and Game than for the Speech sdTheta itself. The increase in Speech words per minute was positively correlated with sdTheta in the Speech (Front: *r*_(16)_ = 0.54, *p* = 0.023), STM (Center: *r*_(16)_ = 0.63, *p* = 0.007), and PVT (Center: *r*_(15)_ = 0.50, *p* = 0.045).

Overall, these results show that behavior and sdTheta can correlate but not necessarily, nor even especially, within the same task. While none of these correlations survive correction for multiple comparisons, the absence of a clear preference for within-task correlations is indicative.

## Discussion

In the literature, there exists two opposing interpretations of theta oscillations: one posits that they reflect cognition, the other that they reflect sleep pressure and possibly even local sleep. With this study, we investigated whether this paradox could be resolved by the existence of separate oscillations in the theta band. Our results clearly indicate that theta caused by sleep deprivation is not strictly a manifestation of classic fmTheta because: (1) their primary sources are in different cortices, namely the right superior frontal gyrus for sdTheta and the left anterior cingulate cortex for fmTheta; and (2) sdTheta is present in a broader subset of areas (Fig. 4).

Despite these differences in sources, we did not find evidence of the simultaneous occurrence of sdTheta and fmTheta during the short-term memory task performed under sleep deprivation (Fig. 5), nor distinct theta peaks in EEG power spectrums (Fig. 11) which would have further supported an interpretation of two independent oscillations. In Vyazovskiy and Tobler (2005), sdTheta in rats was at a lower frequency than the wake hippocampal theta rhythm (5.5 vs 7.5 Hz), with both peaks present during sleep deprivation. This was not replicated in our Game condition where only a single theta peak was present during sleep deprivation, despite a strong, slower, baseline fmTheta (Fig. 6*D*). Rather than a separate, additional spectral peak, it appears that fmTheta itself increased in amplitude with sleep deprivation.

For all other tasks, sdTheta occupied a broad range with multiple peaks (Fig. 11). This can be explained by the different waveforms visually identified (Fig. 13): long steady trains of theta in the Game, and high amplitude irregular short bursts in other tasks. These morphologic differences make the theta trains comparable to occipital alpha bursts, and the short bursts more comparable to isolated slow waves in sleep. This could mean that sleep deprivation in humans induces two types of changes in theta: an increase in fmTheta when already present at baseline, and the appearance of local sleep.

An alternative, simpler explanation is that theta may reflect the same mechanism during both cognition and sleep deprivation, regardless of waveform. Simultaneous EEG-fMRI studies previously found that fmTheta originating from the medial prefrontal cortex corresponds to BOLD deactivations in these areas (Scheeringa et al., 2008, 2009). Our source localization of sdTheta across the different tasks also suggests that these oscillations may be a marker for cortical areas not in use.

First, we found high sdTheta activity in the bilateral (but especially left) supplementary motor area in the Music listening condition. It is compelling that the one task not requiring movement showed such strong theta activity in brain areas involved in complex motor planning (Goldberg, 1985). The PVT also showed strong activity in bilateral supplementary motor areas, which may seem contradictory. However, the PVT required simply pushing a button after a very obvious stimulus appeared; this is a reflexive response with little need for deliberative action. By contrast the LAT, which had identical motor requirements but difficult to detect stimuli, despite otherwise widespread high-amplitude theta, showed less activity in the supplementary motor areas than the PVT (Fig. 9). Supporting this distinction between reflexive and deliberative action, mean reaction times of the LAT were ∼20% slower than during the PVT (Fig. 14*B*,*C*), despite identical task requirements (respond within 0.5 s). Vice versa, supplementary motor area activity did not significantly increase in the Speech or Game, two tasks characterized by deliberative motor control.

Second, high sdTheta was found in the right inferior temporal cortex in the Game, extending all the way to the fusiform gyrus. These areas collectively form the ventral visual pathway responsible for object recognition (Ishai et al., 1999). This is in opposition to the dorsal visual pathway running from the occipital cortex to dorsal parietal areas such as the supramarginal gyrus and parietal sulcus, where object location is processed (Freud et al., 2016). The Game was almost exclusively a spatial task, requiring participants to map out a target path for a bouncing ball. The only other task to show significant theta activity in the inferior temporal cortex was the LAT, a spatial attention task. Instead, the STM, in essence an object recognition task, showed no significant increase in these areas (Fig. 9).

One possible interpretation for theta in unused areas is that it has a role in *active* inhibition. Such a hypothesis has already been proposed for theta during cognition. Buzsáki, in 1996, suggested that theta in the hippocampus could act as a low-energy solution to selective inhibition (Thompson and Best, 1989; Buzsáki, 1996), such that only neurons synchronized to fire at the correct phase of an ongoing oscillation would successfully transmit action potentials. The role of theta phases in inhibition was supported by phase-targeted closed loop stimulation in mice (Siegle and Wilson, 2014). It may therefore be the case that fmTheta and sdTheta in humans also reflect a low-energy active inhibitory state that conflicting brain networks enter to compensate for cognitive load and sleep deprivation, respectively.

Alternatively, theta could reflect passive cortical disengagement. In this scenario, an entire network or brain area ceases to receive inputs, and essentially goes in “standby.” This is comparable to alpha oscillations in visual areas during eyes closed (Kirschfeld, 2005). An interpretation of theta as disengagement, more so than inhibition, would also explain theta activity occasionally found in NREM1 (Santamaria and Chiappa, 1987), at the transition between wake and sleep. In essence, theta as inhibition would be a compensation mechanism for sleep deprivation, whereas theta as disengagement would be a consequence of sleep deprivation, bringing the brain closer to true sleep.

Regardless of whether theta reflects inhibition or disengagement, our behavioral results support the source localization finding that sdTheta occurs primarily in task-irrelevant areas. Despite large changes in performance with sleep deprivation across most outcome measures in the LAT, PVT, and Speech tasks (Fig. 15*A*), these changes were not especially correlated with sdTheta in their respective tasks (Fig. 15*B*). Equal or even larger correlations were found between changes in performance and sdTheta in different tasks (although without surviving FDR correction). Therefore, it is unlikely that the changes in behavior can be attributed to the occurrence of theta oscillations. As it is, these results suggest only a general relationship between the impact of sleep deprivation on performance and on theta.

The most unexpected finding was the decrease in mistakes during the Speech task, and subsequent anti-correlation with sdTheta in almost all tasks and all ROIs. To our knowledge, there is no prior study with tongue twisters during sleep deprivation; however, a study by Tucker et al. (2010) used a verbal fluency task in which participants had to come up with as many words as possible starting with a specific letter. The authors found both a practice effect and a sleep deprivation effect, such that both improved performance. While we cannot dissociate these effects in our data, we do see that of the four participants who did the baseline session after the sleep deprivation, two still showed notably higher performance during SD compared with BL, and two showed no change (Extended Data Fig. 14-1*D*). It is therefore possible that this speech task also improves with both repetition and sleep deprivation. A possible explanation could be that the more “sleep deprived” prefrontal control areas are, the less inhibited participants, especially non-native speakers, become. Alternatively, given that sdTheta is hypothesized to reflect plasticity and therefore ability to learn, the same interindividual differences in changes in theta with time awake could be reflected as individual differences in tongue-twister learning ability. More studies investigating the link between sdTheta and learning are needed to resolve this problem.

While our study offers unique insight into theta under different conditions, it also suffers limitations. First, the sessions were not conducted in counterbalanced order. While previous studies (Hung et al., 2013; Bernardi et al., 2015) have demonstrated sdTheta returns to baseline following recovery sleep, it is still possible that some of the effects we observe (e.g., disappearance of fmTheta with sleep deprivation) are a consequence or at least an interaction with task repetition. Furthermore, caution is needed when interpreting the source localization data, given the lack of structural MRIs and digitization of electrode positions. Finally, there are many other factors that can influence theta (fatigue, age, etc.), and fmTheta is not even the only manifestation of theta during cognition within a single task (Pastötter and Bäuml, 2014; Brzezicka et al., 2019). These results therefore cannot be generalized beyond classic frontal-midline theta as recorded from surface EEG. It is imperative to verify and expand these results with other experiments, analyses, and participant populations.

In conclusion, we do not provide a definitive resolution to the theta paradox but suggest three possible explanations for our results: (1) fmTheta and sdTheta are separate oscillations, but both can occur during sleep deprivation, maybe one as a compensation mechanism, the other as local sleep; (2) sdTheta is merely a more widespread form of fmTheta, and both reflect active cortical inhibition of task-irrelevant networks; (3) or both reflect passive cortical disengagement.
