# Supplementary figures and images for: The Theta Paradox: 4-8 Hz EEG Oscillations Reflect Both Sleep Pressure and Cognitive Control
Source: J Neurosci. 2022 Nov 9;42(45):8569–86. doi: 10.1523/JNEUROSCI.1063-22.2022 (PMC9665934; doi:10.1523/JNEUROSCI.1063-22.2022)

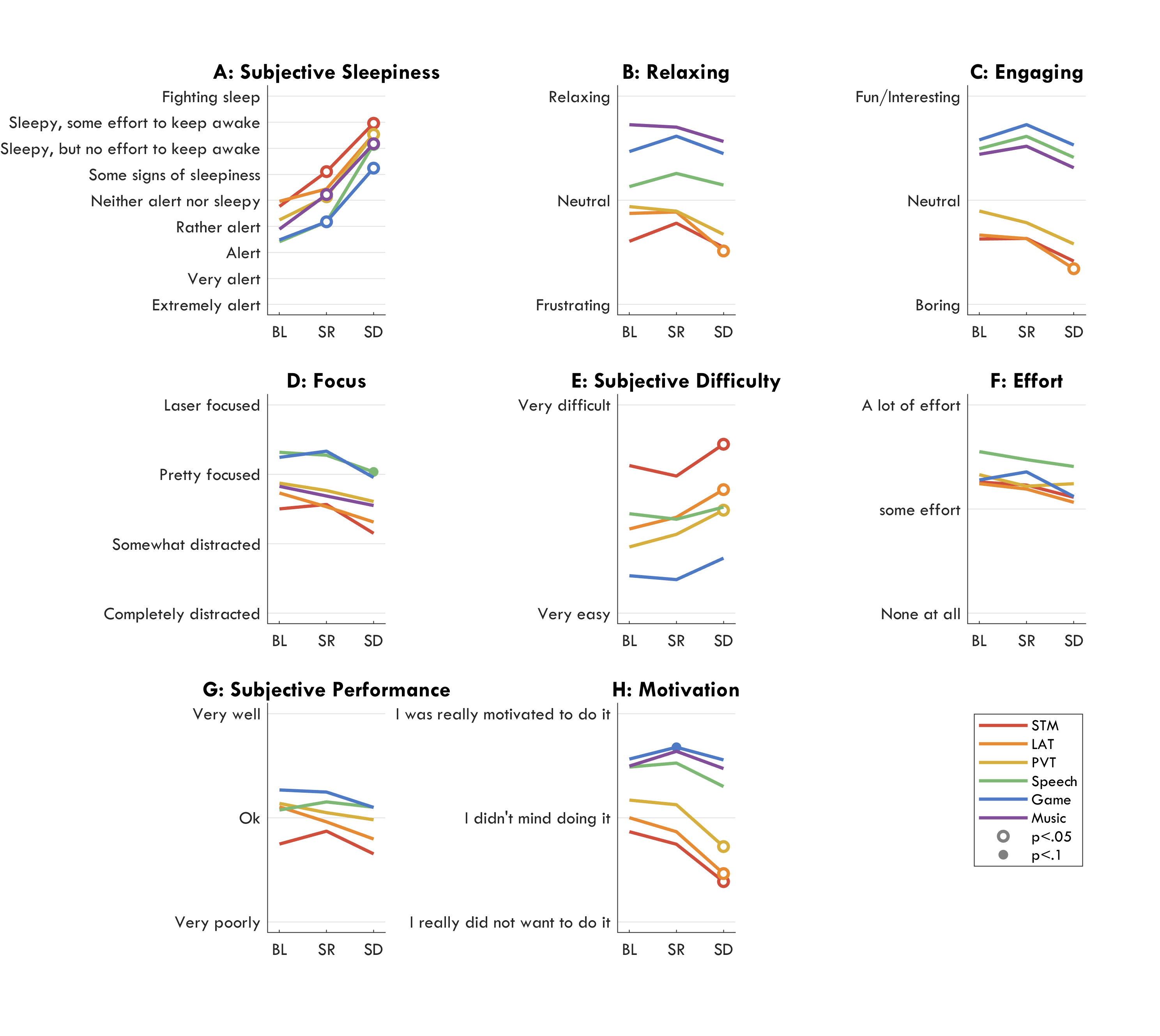

Supplement: Extended Data Figure 3-1 — Questionnaire answers for each task. All answers were given on a ∼10-cm slider with labels at specific intervals (indicated on the y-axis). White-filled circles indicate a significant change from BL, small colored circles a trend, FDR corrected. Each question was asked in this order. A two-way rmANOVA was conducted for each question with factors session, task, and their interaction. Due to occasional missing data, not all analyses included every participant, therefore for each statistic, the degrees of freedom (subscript in F(A,B)) indicate the sample size (N=BA+1). No analysis had fewer than 16 participants, and the majority had all 18. A, “Please indicate your sleepiness right now.” (Session: F(2,30) = 35.42, p < 0.001, η2 = 0.35; Task: F(5,75) = 14.7, p < 0.001, η2 = 0.073; Interaction: F(10,150) = 0.96, p = 0.440, η2 = 0.008). B, “How did you experience this task? [Relaxing].” (Session: F(2,30) = 5.13, p = 0.012, η2 = 0.019; Task: F(5,75) = 41.97, p < 0.001, η2 = 0.530; Interaction: F(10,150) = 1.11, p = 0.365, η2 = 0.010). C, “How did you experience this task? [Engaging].” (Session: F(2,30) = 6.42, p = 0.015, η2 = 0.027; Task: F(5,75) = 43.63, p < 0.001, η2 = 0.548; Interaction: F(10,150) = 1.52, p = 0.191, η2 = 0.010). D, “How focused on the task were you?” (Session: F(2,30) = 5.25, p = 0.016, η2 = 0.039; Task: F(5,75) = 13.63, p < 0.001, η2 = 0.235; Interaction: F(10,150) = 0.39, p = 0.853, η2 = 0.006). E, “How hard was it to perform this task?” (Session: F(2,30) = 7.02, p = 0.006, η2 = 0.038; Task: F(4,60) = 31.67, p < 0.001, η2 = 0.426; Interaction: F(8,120) = 0.71, p = 0.585, η2 = 0.008). F, “How much effort did you put into performing this task? (Think about how much you tried to do well, and how much more you could have done).” (Session: F(2,28) = 2.17, p = 0.139, η2 = 0.022; Task: F(4,56) = 4.84, p = 0.015, η2 = 0.114; Interaction: F(8,120) = 0.37, p = 0.838, η2 = 0.006). G, “How well do you think you did the task?” (Session: F(2,30) = 2.02, p = 0.156, [file ns-JN-RM-1063-22-s01.tif]

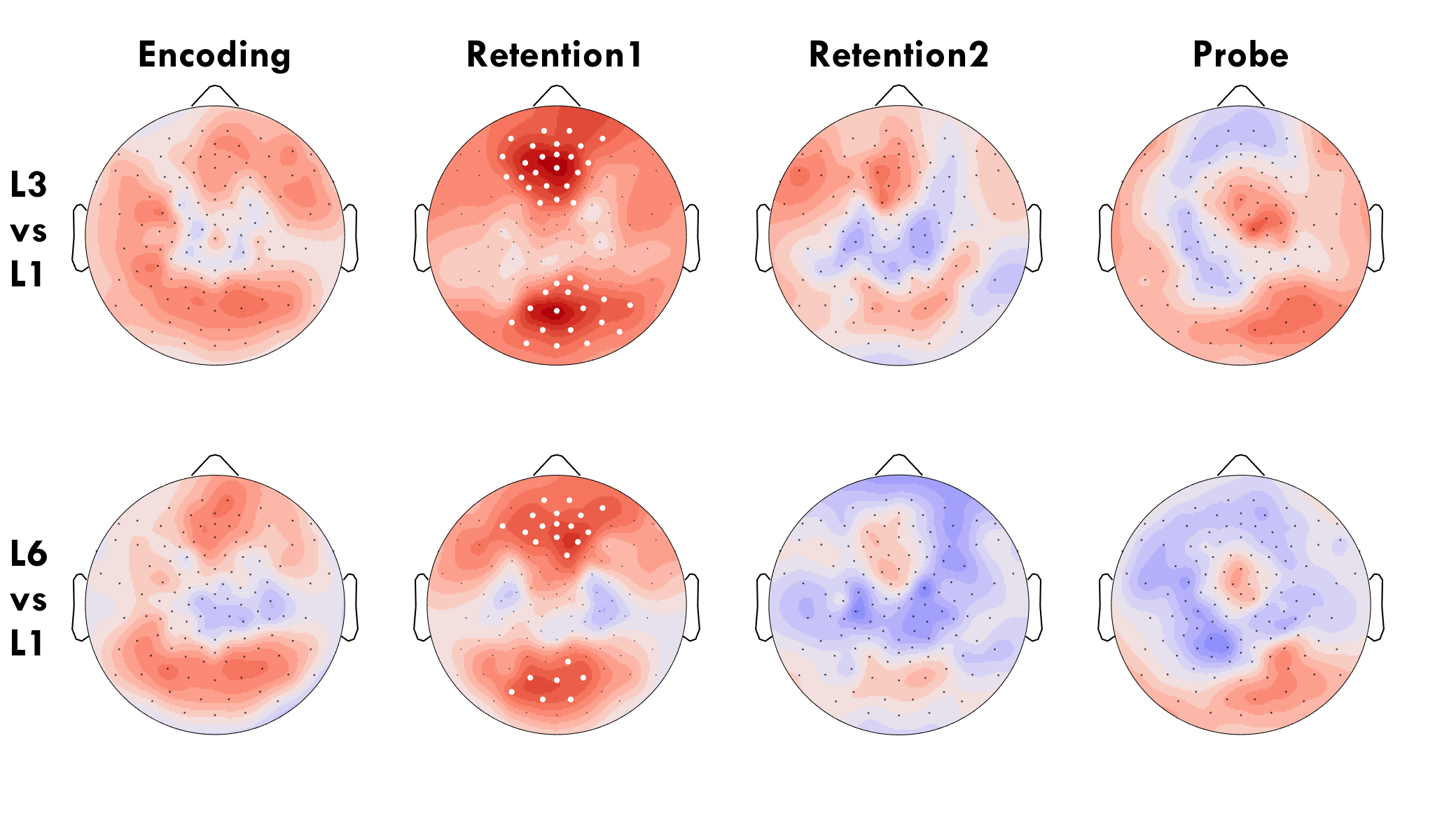

Supplement: Extended Data Figure 4-1 — Difference in theta power topographies between levels at BL for every epoch of the STM task. Level 1 is compared to level 3 (top row) and level 6 (bottom row) for each 2-s epoch. Encoding was when participants were viewing the items to memorize. Retention1 is the first half of the window in which participants had to hold the items in memory. Retention2 is the second half. Probe is when participants had to indicate whether a probe symbol was part of the original set. Participants' answers ended the probe window; therefore, this 2-s epoch could also encompass some of the rest window that followed. The color scale is the same as in Figure 4, with red indicating an increase in theta relative to L1. Download Figure 4-1, TIF file. [file ns-JN-RM-1063-22-s02.tif]

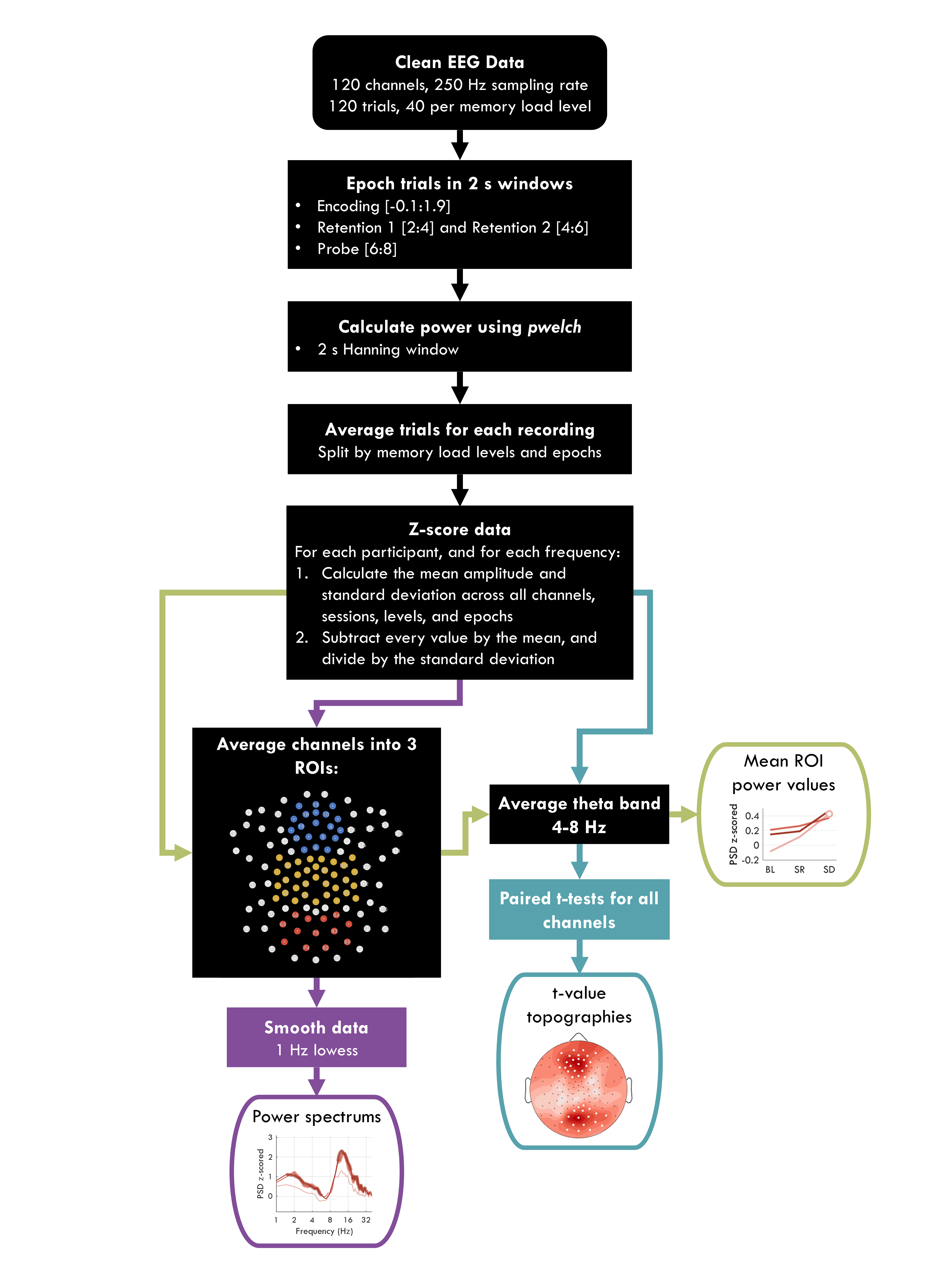

Supplement: Extended Data Figure 5-1 — STM EEG analysis pipeline. Starting data was preprocessed as described in Extended Data Figure 6-1. Sections in black indicate steps in common between more than one analysis. Purple indicates steps for calculating power spectrums, teal indicates steps specific to topographies, and green steps for average region of interest (ROI) power. The ROI channels were preselected, and in the diagram the Front ROI is in blue (3, 4, 5, 6, 9, 10, 11, 12, 13, 15, 16, 18, 19, 20, 22, 23, 24, 112, 118, 124), Center in yellow (7, 30, 31, 35, 36, 37, 41, 42, 47, 51, 52, 53, 54, 55, 60, 61, 62, 78, 79, 80, 85, 86, 87, 92, 93, 97, 98, 103, 104, 105, 106, 110, 129), and Back in red (65, 66, 69, 70, 71, 74, 75, 76, 82, 83, 84, 89, 90). STM epochs were all of 2 s in duration; however, the encoding epoch was shifted 0.1 s earlier to avoid initial retention EEG responses. The pipeline for all other task EEG analyses (Fig. 7) is identical, except without trials or epoching, and using 8-s windows with 75% overlap across the first 4 min of data. Download Figure 5-1, TIF file. [file ns-JN-RM-1063-22-s03.tif]

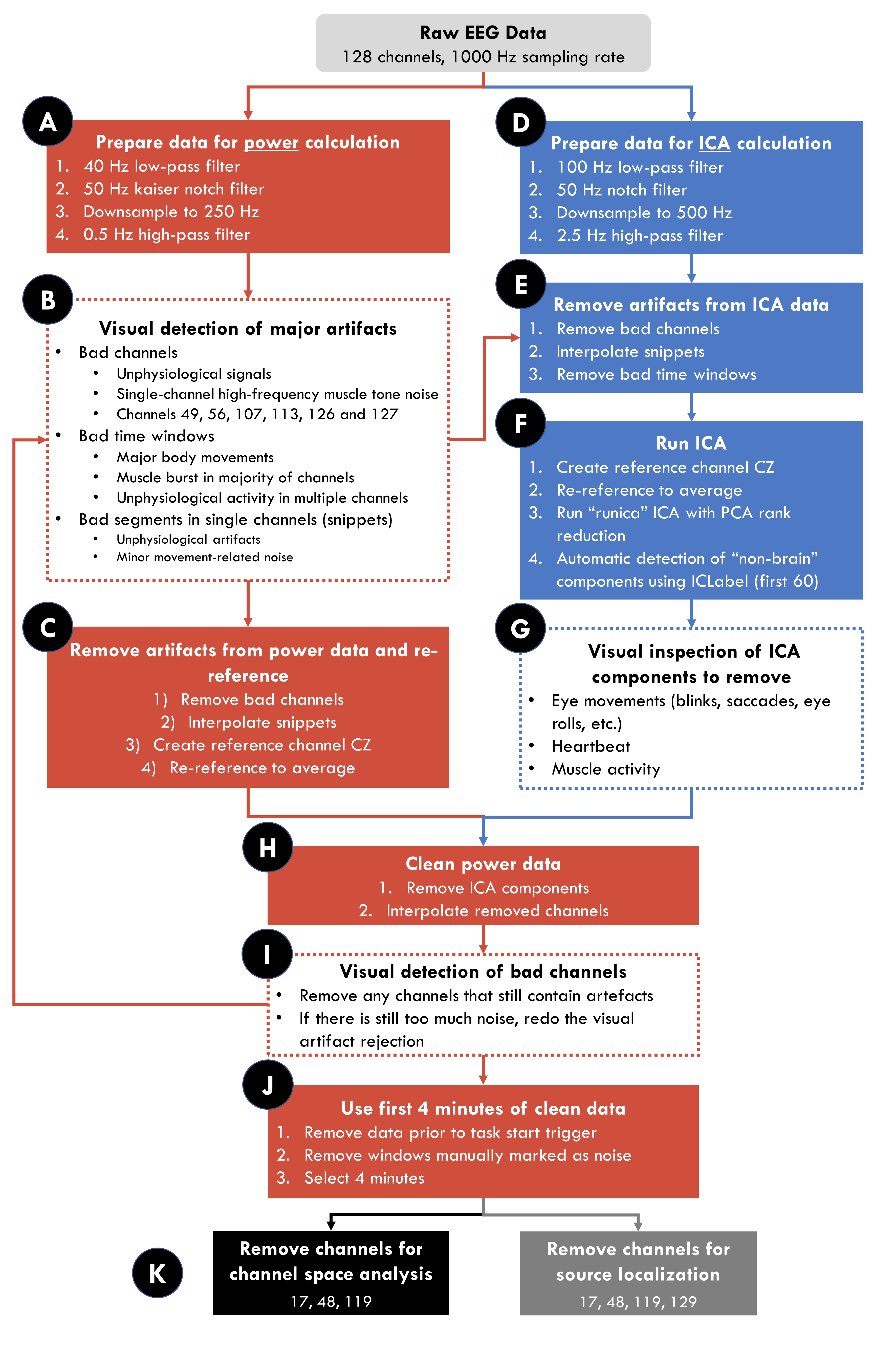

Supplement: Extended Data Figure 6-1 — Pipeline for data preprocessing. Red steps were conducted on the data used for the power analysis (250-Hz sampling rate, 0.5–40 Hz). Blue steps were conducted on the data used for the independent component analysis (ICA; 500-Hz sampling rate, 2.5–100 Hz). White-filled steps (B, G, I) involved manual work. A, First, data was low-pass filtered at 40 Hz using EEGLAB's default filter. A Kaiser notch filter was then applied to remove 50-Hz line noise and subsequent harmonics. Data was then down-sampled to 250 Hz. A 0.5-Hz high-pass Kaiser-window-based FIR filter was then applied (0.25-Hz stopband, 60-dB stopband attenuation, 0.05 passband ripple). B, The data were visually inspected to identify bad channels (Extended Data Fig. 6-2A), bad time windows, and bad single-channel segments (i.e., snippets). Bad channels were considered as such that if they contained any nonphysiological signals (anything not from the brain, muscles, or eyes) that occurred either continuously or repeatedly throughout the recording. Furthermore, external channels outside the EGI net were automatically removed (49, 56, 104, 113), as well as the face channels (126, 127). Bad time windows were any segments in time in which an artifact affected multiple channels at once, often due to body movements or brief muscle clenching. Bad snippets were nonphysiological artifacts affecting only a few channels. C, Prior to removing artifacts with ICA, bad channels were removed, snippets interpolated, and the data re-referenced to the average. However, bad time windows were not removed. These are removed later (J). D, Data used for calculating the ICA were filtered and downsampled differently from A to maximize the detection of eye-movement artifacts. E, F, For ICA, only clean data were used, Cz was restored, and all channels re-referenced to the average. EEGLAB's “runica” ICA algorithm was applied, with principal component analysis (PCA) rank reduction. Using EEGLAB's ICLabel function (v1.2.4), the first 60 comp [file ns-JN-RM-1063-22-s04.tif]

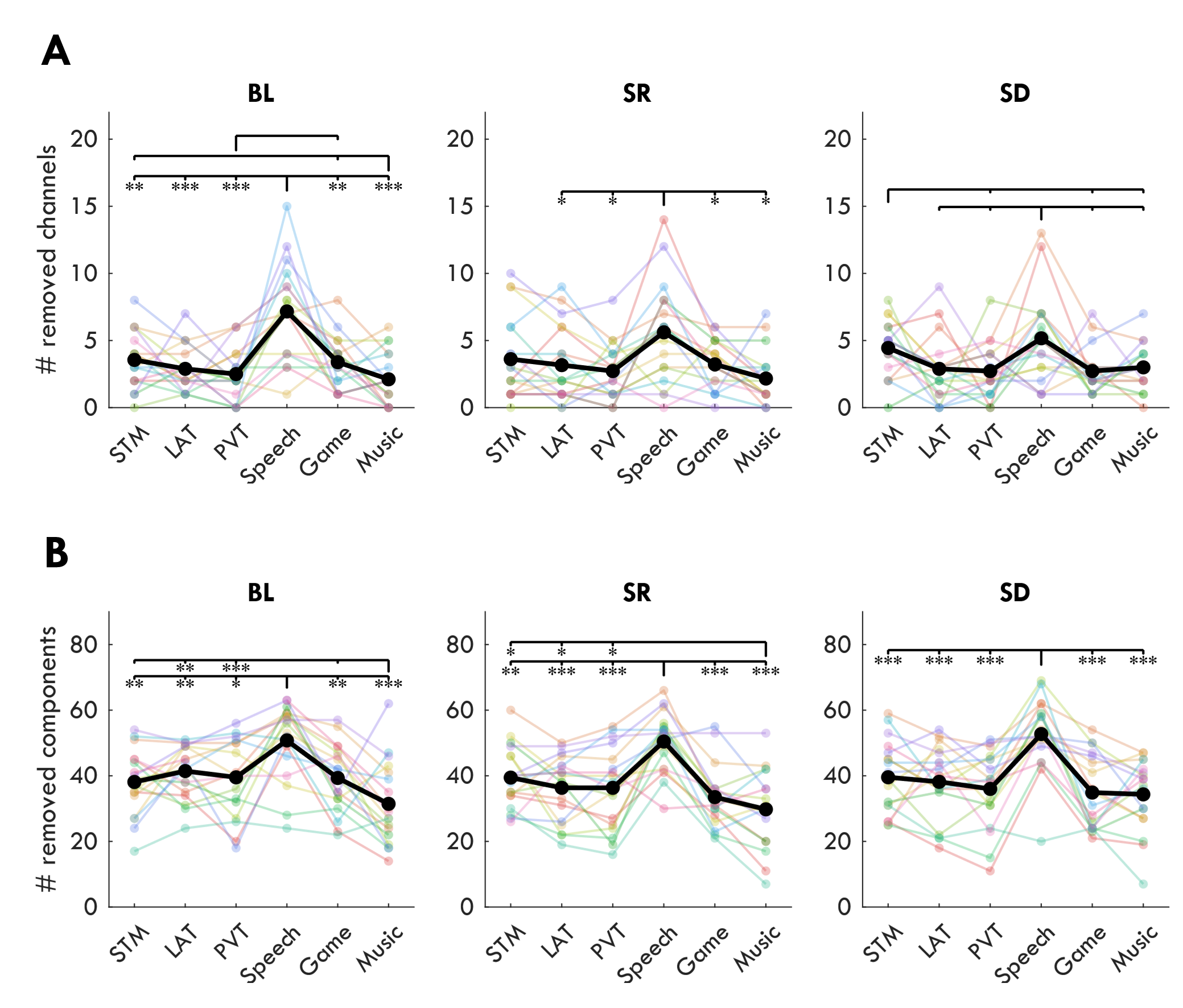

Supplement: Extended Data Figure 6-2 — Removed channels and components for each recording and each session. A, Number of channels removed (out of 120). Each colored line represents a participant. The black line is the group average. B, Number of removed components after ICA (max 120). Asterisks indicate significant differences from paired t tests between sessions, FDR corrected, such that *p < 0.05, **p < 0.01, ***p < 0.001. Download Figure 6-2, TIF file. [file ns-JN-RM-1063-22-s05.tif]

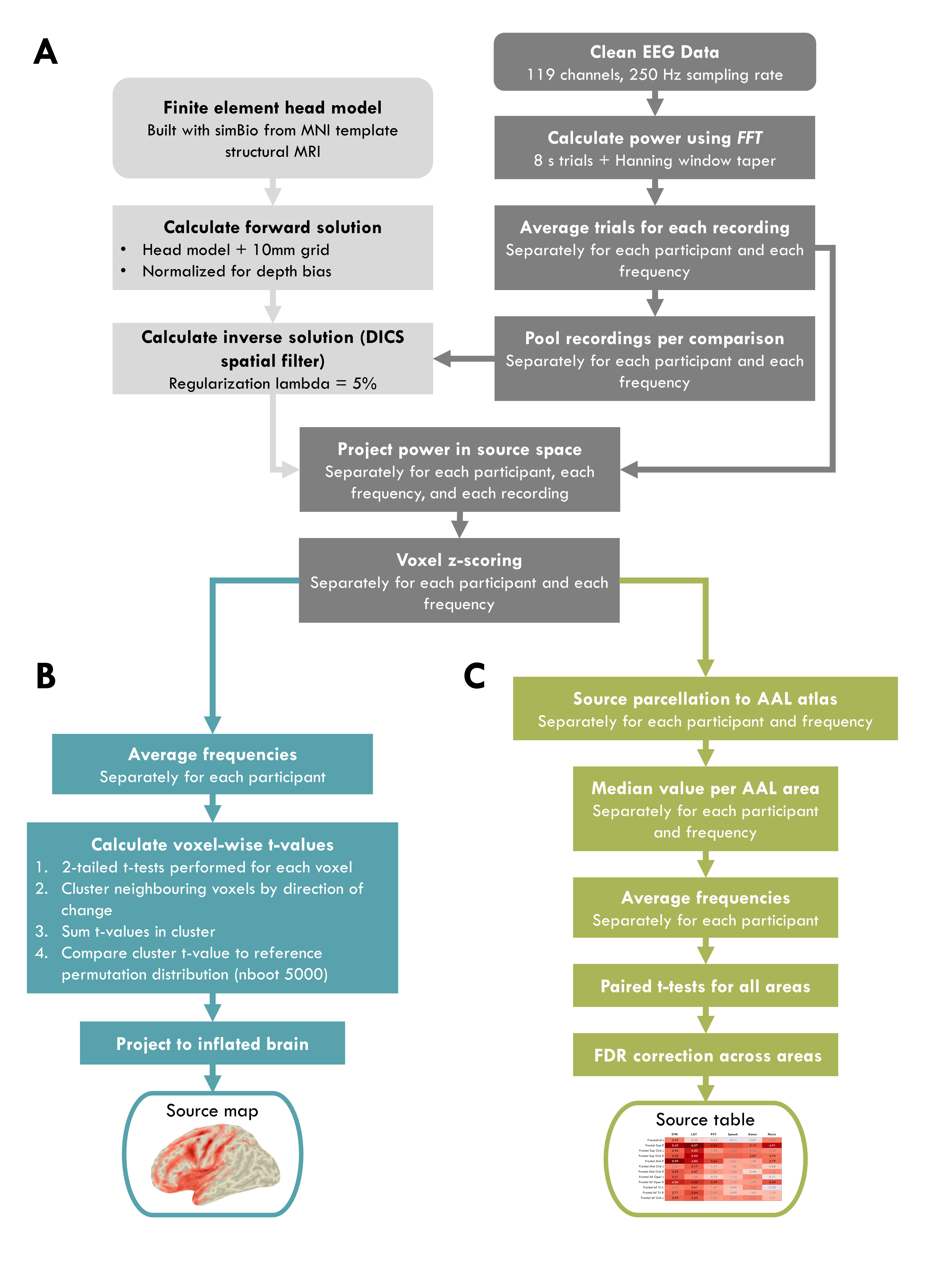

Supplement: Extended Data Figure 8-1 — Pipeline for source localization analysis. A, To compute theta power for the source localization, we used a fast Fourier transform (FFT) with a Hanning taper, applied to each 8-s window. To construct the forward model, we obtained a finite-element head model, implemented with the SimBio toolbox based on the segmentation of the template T1 MRI image from the Montreal Neurological Institute into gray matter, white matter, cerebrospinal fluid, scalp, and skull. Subsequently, a standard 3D grid (10-mm spacing, 3294 voxels inside the head) and the head model were used to compute the leadfield matrix. To avoid depth bias, the leadfield was normalized. As an inverse solution, we used the DICS beamformer technique. We first computed common spatial filters based on a cross-spectral density matrix obtained from pooled conditions, with a regularization parameter lambda set to 5%. The precomputed common spatial filters were then applied independently to each condition. After projecting each recording to the source space, each frequency was z-scored for each participant (as done in the channel space). B, 3D brain source maps. Nonparametric cluster correction was implemented instead of FDR because it was only intended as a mask for the inflated brains plots, and not as hypothesis testing. First, independent samples t tests for all voxels were done for the contrast of interest (two-tailed, p < 0.05). Next, significant neighboring voxels were clustered if they showed the same direction of effect. To assess the statistical significance of each cluster, a cluster-level test statistic was calculated by computing the sum of all t values in the cluster. The significance of each cluster was estimated by comparing the cluster-level test statistic to a reference permutation distribution derived from the data. The reference distribution was obtained by randomly permuting the data 5000 times. The cluster p-value was estimated as the proportion of the elements in the reference distribution ex [file ns-JN-RM-1063-22-s06.tif]

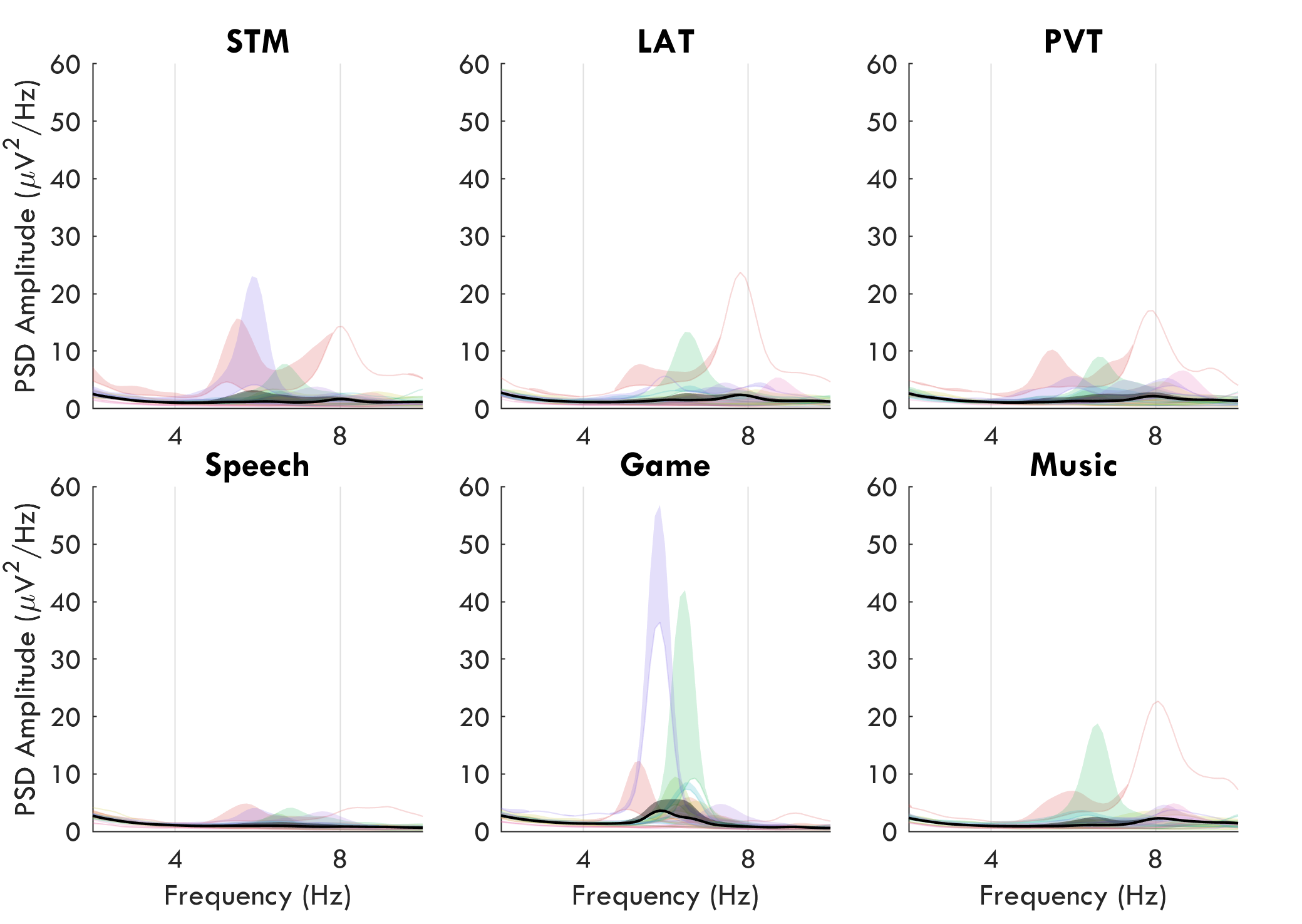

Supplement: Extended Data Figure 11-1 — Uncorrected power spectrums from the front ROI for each task. Overlapping EEG power spectrums, untransformed, from the Front ROI of each task for every participant. The base curve of each colored patch represents the BL spectrum, the upper curve represents the SD spectrum, and the filled-in area reflects the increase in power. The average power change is the final patch in black. Download Figure 11-1, TIF file. [file ns-JN-RM-1063-22-s07.tif]

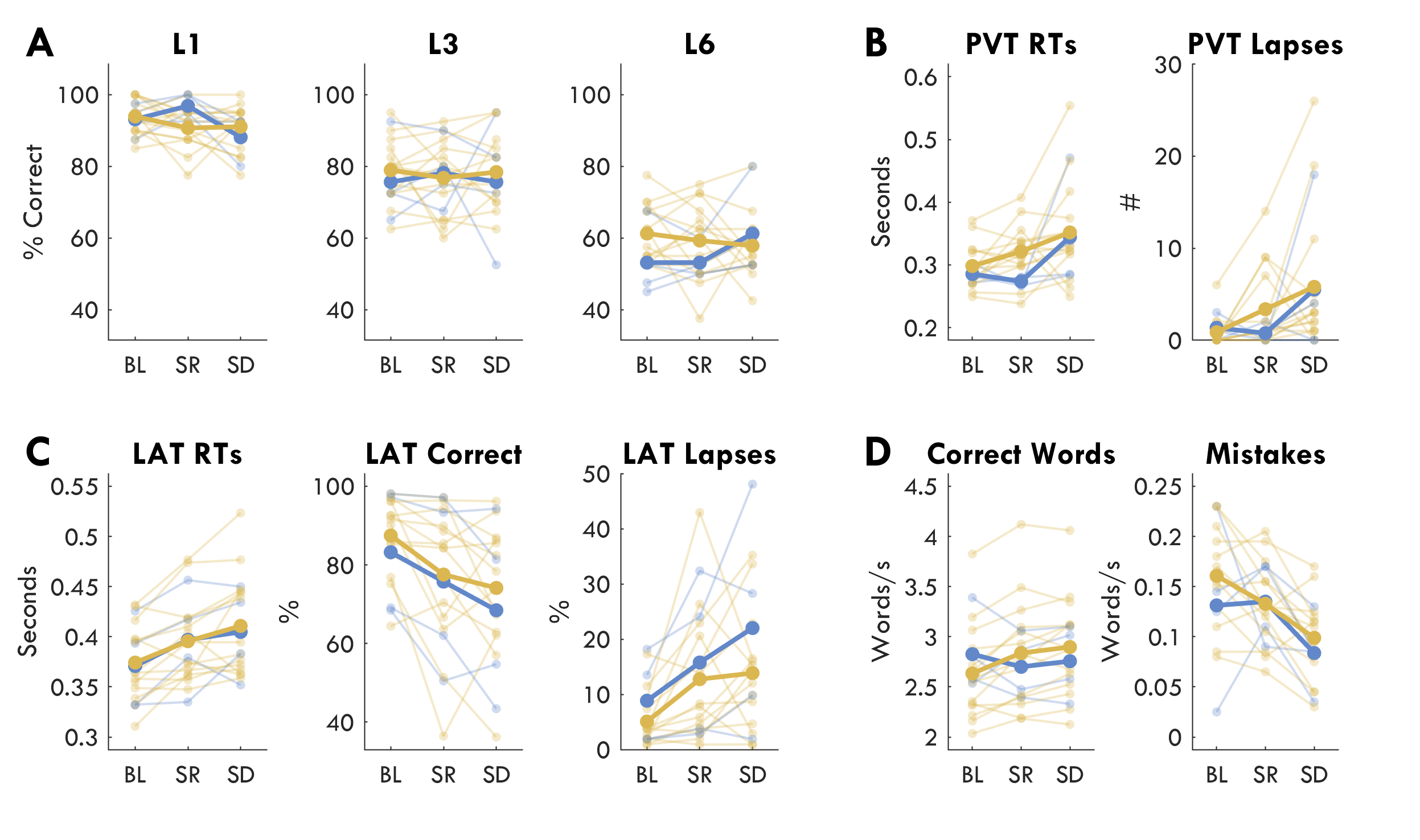

Supplement: Extended Data Figure 14-1 — Task performance by experiment order. Same data as Figure 14. Participants who conducted the baseline night after the sleep deprivation session are highlighted in blue (N = 4), the remainder are in yellow (N = 14). Download Figure 14-1, TIF file. [file ns-JN-RM-1063-22-s08.tif]
